# All Platinum(II) Ions Are Equal, but Some React Differently: Insights Into the Biomolecular Reactivity of Platinum(II)‐Based Anticancer Agents

**DOI:** 10.1155/bca/9865583

**Published:** 2026-08-20

**Authors:** Andrea Cucchiaro, Monika Cziferszky

**Affiliations:** ^1^ Institute of Pharmacy, Pharmaceutical Chemistry, Center for Molecular Biosciences Innsbruck (CMBI), University of Innsbruck, Innrain 80/82, Innsbruck A-6020, Austria, uibk.ac.at

## Abstract

Platinum‐based anticancer agents are widely used in cancer therapy due to their efficacy in killing cancer cells through interactions with the DNA. However, their clinical application is limited by severe side effects and the development of drug resistance, which have been related to their lack of specificity and mechanism of action. To overcome these challenges, novel platinum(II) complexes, K[Pt(Butene‐ASA)Cl_3_] (Compound **1**) and [Pt(L‐Ala)(Butene‐ASA)Cl] (Compound **2**), were previously synthesized and characterized. Particularly, Compound **2** showed cytotoxic activity comparable to that of cisplatin (DDP) against all tested cancer cell lines in a previous study. The current investigation employed electrospray ionization mass spectrometry (ESI‐MS) to analyze the interactions of these complexes with model biomolecules, including an oligonucleotide (8mer), peptide (Angiotensin I, AT1), and protein (Cytochrome c, CytC). DDP and oxaliplatin (OxPt) were used as reference substances. Tandem mass spectrometry (MS/MS) and UV‐Vis spectroscopy enabled the identification of the platination sites on the model biomolecules. Compound **2** demonstrated lower reactivity toward DNA than DDP, while both compounds **1** and **2** exhibited a significant shift in reactivity toward peptides and proteins, particularly compared to DDP and OxPt. The results suggest that Zeise’s salt derivatives may preferentially target peptides and proteins rather than DNA, which could provide a novel mechanism of action for platinum‐based anticancer agents. These findings expand the understanding of platinum(II) complex reactivity and highlight their potential for developing alternative therapeutic strategies aimed at overcoming the limitations of traditional platinum‐based drugs.

## 1. Introduction

Platinum‐based compounds have been widely used as anticancer agents since the clinical approval of cisplatin (DDP) in 1978 and remain among the most important chemotherapeutic drugs despite advances in precision medicine and immunotherapy, serving as a standard therapy for several malignancies [1]. Additionally, there is also a resurgence of interest in this regard, driven by improved understanding of cancer’s cellular mechanisms. In fact, recent advances in elucidating the mechanisms underlying efficacy, resistance, toxicity, and immunomodulatory effects are reshaping our understanding of the biological activity of these compounds. In parallel, the identification of non‐DNA molecular targets is uncovering previously unrecognized mechanisms of action. Furthermore, the integration of advanced preclinical models, rational combination strategies, and innovative delivery platforms is expanding the therapeutic landscape of Pt‐based anticancer agents [1, 2].

Despite their remarkable success, the efficacy of these chemotherapeutics is often compromised by severe, dose‐limiting side effects—such as nephrotoxicity, hepatotoxicity, cardiotoxicity, and ototoxicity [3, 4]. Moreover, the emergence of intrinsic or acquired resistance further undermines clinical outcomes [5]. Over the past decades, medicinal chemists worldwide have pursued various strategies to address these challenges. One of those strategies consists of developing new platinum‐based anticancer agents with a different molecular target. This idea led to the synthesis of derivatives of Zeise’s salt incorporating a modified acetylsalicylic acid ligand, with the aim of obtaining cytotoxic cyclooxygenase‐2 (COX‐2) inhibitors (e.g., K[Pt(Butene‐ASA)Cl_3_], (**1**)) [6–8]. The coordination compounds included in this group exhibited promising IC_50_ values, but showed moderate stability in aqueous solutions and high reactivity in the presence of sulfur‐containing biomolecules [9]. With the aim of addressing these limitations, two chlorido ligands were replaced with a chelating amino acid, namely, L‐alanine, to stabilize the labile ligand in the *trans* position to the olefinic ligand ([Pt(L‐Ala)(Butene‐ASA)Cl], (**2**)) [10]. This platinum(II) complex not only demonstrated enhanced stability in aqueous solutions, improved solubility in water, and reduced reactivity toward sulfur‐donor compounds [11], but also showed cytotoxic activity comparable to that of DDP.

However, it was not possible to establish a correlation between the COX‐2 inhibiting ability of these complexes and their cytotoxic activity, suggesting a different molecular target for these platinum‐based anticancer agents.

Electrospray ionization mass spectrometry (ESI‐MS) is a well‐established technique for the analysis of the interactions of metallodrug candidates with target molecules [12], such as oligonucleotides [13], peptides [14], and proteins [15], on account of its high sensitivity and compatibility with biomolecules.

The mild ionization conditions of ESI‐MS enable the detection of noncovalent and covalent adducts, which are typically produced during the reaction of coordination compounds with biomolecules. Structural information, including metalation sites on biomolecules, can be obtained by tandem mass spectrometry (MS/MS), where adducts formed by the interaction of biomolecules with coordination compounds can be isolated and subjected to various amounts of collision energy. Analysis of the resulting fragments gives insight into binding motives and donor atoms. This top‐down approach offers significant advantages over the enzymatic digestion‐based bottom‐up approach, as it eliminates the need for complex sample preparation and minimizes the risk of metal ion cleavage from the biomolecule.

**SCHEME 1:**
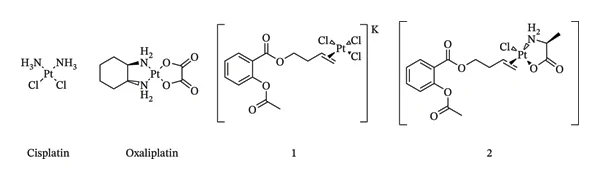
Structure of the platinum(II) complexes involved in the current study.

In this study, we compare the reactivity profiles of approved platinum‐based anticancer drugs, DDP and oxaliplatin (OxPt), with two Zeise’s salt derivatives: **1** and **2** (Scheme 1).

To investigate the interaction of these compounds with DNA, we selected the oligonucleotide ATTGGCAC (8mer) as a model, which has been shown to be effective for studying the interactions of metallodrugs with DNA before [16]. Notably, this specific sequence allows DDP and OxPt to form adducts involving the two adjacent guanine bases exclusively, according to the coordination mode reported in the literature [3, 17].

To complement the DNA model, we selected a peptide and a protein based on specific criteria. These biomolecules needed to be commercially available, water‐soluble, and highly stable in solution. Furthermore, they needed to be easily ionizable, making them suitable for ESI‐MS investigations, and their structures had to be previously characterized by X‐ray diffractometry or NMR spectroscopy.

Angiotensin I (AT1) was chosen as the peptide model due to its proven utility in previous studies of Zeise’s salt derivatives. This is primarily due to the presence of two histidine residues in its sequence, which, together with the N‐terminus, represent potential platination sites [9, 18–20].

Cytochrome c (CytC) was selected as the protein model, owing to its well‐recognized value as a model protein for organometallic interaction studies [12, 15, 21–25]. CytC is a water‐soluble protein that features a heme group anchored via two cysteine residues. With a molecular weight of approximately 13 kDa, CytC plays a key role in cellular respiration by participating in the electron‐transport chain within the mitochondria [26, 26].

The results of this study aim to contribute to a better understanding of the mechanism of action of these novel platinum complexes and support the development of alternative therapeutic strategies.

## 2. Materials and Methods

Deionized water was prepared using a Millipore Milli‐Q Gradient A10 Water Purification system (Merck Millipore, Billerica, MA, USA). Positive‐mode high‐resolution ESI‐MS (HR‐ESI‐MS) analysis was conducted using an Orbitrap Elite mass spectrometer (Thermo Fisher Scientific, Waltham, MA, USA), whereas negative‐mode HR‐ESI‐MS analysis was conducted using an Orbitrap Exploris 240 (Thermo Fisher Scientific, Waltham, MA, USA). Reported m/z values correspond to the most intense signal within the isotopic cluster of the complexes, corresponding to species containing ^195^Pt and, where relevant, ^35^Cl.

The oligonucleotide with the sequence ATTGGCAC (8mer) was prepared and provided by Microsynth, Switzerland. Formic acid (FA), AT1 human acetate salt hydrate, and CytC from equine heart were supplied by Sigma‐Aldrich and used as received. Cis‐diaminodichloroplatinum(II) (Cisplatin, DDP) and oxalato(trans‐l‐1,2‐cyclohexanediamine)platinum(II) (OxPt) were purchased from BLDpharm and used as received.

K[Pt(Butene‐ASA)Cl_3_] (**1**) and [Pt(L‐Ala)(Butene‐ASA)Cl] (**2**) were prepared following the procedures reported in the literature [7, 10].

### 2.1. Incubation Reaction of 8mer With Metallodrugs

A stock solution of 8mer (100 μM) was prepared by dissolving the oligonucleotide in water. Stock solutions of the metallodrugs (10 mM) were prepared by dissolving DDP and OxPt in Milli‐Q water, and Compounds **1** and **2** in methanol.

Aliquots of 300 μL of the 8mer stock solution were combined with 9 μL of each platinum coordination compound, yielding four different reaction mixtures. The molar ratio of platinum compound to oligonucleotide was 3:1. The reaction mixtures were incubated at 37°C with gentle shaking at 200 rpm for 48 h.

### 2.2. Sample Preparation for the Reaction Between 8mer and Metallodrugs

60 μL of the reaction mixture were collected after 3, 6, 24, and 48 h and diluted with 120 μL of Milli‐Q water and 120 μL of a 50:50 methanol–isopropanol mixture. The samples were filtered (0.22 μm pore size) prior to analysis.

### 2.3. Incubation Reaction of AT1 and CytC With Metallodrugs

A stock solution of the peptide or protein (1 mM) was prepared in Milli‐Q water. Stock solutions of the metallodrugs (10 mM) were obtained by dissolving DDP or OxPt in Milli‐Q water and Compounds **1** and **2** in methanol. 30 μL of each metallodrug stock solution was mixed with 100 μL of the peptide/protein stock solution and diluted with 870 μL of Milli‐Q water (final peptide/protein concentration = 100 μM; the molar ratio of platinum compound to peptide/protein was 3:1). The reactions were stored under the same conditions as those used for the 8mer experiment.

### 2.4. Sample Preparation for the Reaction Between AT1 and Metallodrugs

60 μL of the reaction mixture was collected after 3, 6, 24, and 48 h and diluted with 120 μL of Milli‐Q water and 120 μL of a 50:50 methanol–isopropanol mixture. The samples were filtered (0.22 μm pore size) prior to analysis.

### 2.5. Sample Preparation for the Reaction Between CytC and Metallodrugs

60 μL of the reaction mixture was collected after 3, 6, 24, and 48 h and diluted with 120 μL of Milli‐Q water and 120 μL of a 50:50 methanol–isopropanol mixture containing 0.1% FA. A blank incubation containing only CytC in Milli‐Q water was prepared and analyzed at each time point as a control. The samples were filtered (0.22 μm pore size) prior to analysis.

### 2.6. ESI‐MS Analysis

Samples were analyzed using an Orbitrap‐equipped MS in positive or negative mode under standard operating conditions using the HESI source (heated ESI) and the syringe pump. Higher energy collisional dissociation (HCD) experiments were performed manually on all ions of interest using an isolation window of 7 m/z. The normalized collision energy (NCE) was increased stepwise.

Data were analyzed using Xcalibur software and selected adducts analyzed with the Apm^2^S (for peptides and proteins) [27] or Aom^2^S (for oligonucleotides) [28] software tools to determine the binding site. Search parameters are provided in Table S16. The fragments are named according to the nomenclature proposed by Mcluckey et al. [29] and Chu et al. [30] for oligonucleotides and peptides, respectively. Experimental and calculated m/z values for the fragments refer to the highest signal in the isotopic cluster (Figure S8). Molecular formulas and sequence information can be found in Tables S1 and S2.

## 3. Results and Discussion

### 3.1. Adduct Formation and Speciation

#### 3.1.1. Oligonucleotide

We investigated the reactivity of the Pt‐based anticancer agents toward a model oligonucleotide (8mer). As the phosphate groups forming the backbone of the oligonucleotide are deprotonated at neutral pH, we recorded all mass spectra of oligonucleotides in the negative mode, considering the model as a hepta‐negative molecule ([8mer]^7−^). A blank sample of the 8mer was analyzed to confirm the oligonucleotide’s identity and to exclude the presence of double‐stranded adducts (see Table S3).

Each platinum‐based coordination compound was incubated with the model oligonucleotide in a molar ratio of 3:1 to promote the formation and detection of platinated adducts, which were typically observed in the charge states −3 to −5 (Tables S3–S7). For better clarity, the mass spectra are presented as deconvoluted spectra and the adducts discussed as neutral species.

The reaction of the 8mer with DDP exhibited quick formation of platinated oligonucleotide adducts (Figure S1, Table S4). After 3 h, the signal for unmodified 8mer has vanished completely, and the mono‐platinated adduct [8mer + Pt(NH_3_)_2_] dominated the spectrum. Additionally, di‐ ([8mer + 2 Pt(NH_3_)_2_Cl], [8mer + Pt(NH_3_)_2_Cl + Pt(NH_3_)_2_], and [8mer + 2 Pt(NH_3_)_2_]) and tri‐platinated adducts ([8mer + 3 Pt(NH_3_)_2_Cl] and [8mer + 2 Pt(NH_3_)_2_Cl + Pt(NH_3_)_2_]) were observed. The mono‐platinated adduct got consumed over time and [8mer + Pt(NH_3_)_2_Cl + Pt(NH_3_)_2_] progressively converted to [8mer + 2 Pt(NH_3_)_2_]. Additionally, [8mer + 2 Pt(NH_3_)_2_Cl + Pt(NH_3_)_2_] was converted into [8mer + Pt(NH_3_)_2_Cl + 2 Pt(NH_3_)_2_] and, ultimately, into [8mer + 3 Pt(NH_3_)_2_]. These results are in agreement with the widely reported mode of action of DDP [4], as well as previous similar MS studies [16].

The reaction between the 8mer and OxPt proceeded with slower kinetics compared to the DDP reaction (Figure S2, Table S5). During the first 24 h, the base peak was representative for the unmodified 8mer. Two low‐abundant signals were detected, corresponding to the mono‐platinated species [8mer + Pt(DACH)(Ox)] and [8mer + Pt(DACH)]. After 48 h, however, the latter represented the base peak of the spectrum and other di‐platinated adducts were observed, such as [8mer + 2 Pt(DACH)(Ox)], [8mer + Pt(DACH) + Pt(DACH)(Ox)], and [8mer + 2 Pt(DACH)]. In contrast to DDP, tri‐platinated adducts were barely detectable. These results are also in line with the reported interactions of OxPt with DNA [17], confirming the suitability of the 8mer as a DNA model for metallodrug binding studies.

The reduced ability of OxPt to form adducts with DNA has been previously reported by several studies [17, 31]. OxPt undergoes an activation step in the plasma via exchange of the Ox ligand for two chlorido ligands, which is induced by the physiological concentration of chloride in blood [17, 32]. The dichlorido derivative exhibits enhanced reactivity toward DNA in comparison with the parental complex OxPt [17, 33]. Since we refrained from using chloride salts for our mass spectrometry‐based study, we are likely observing slower kinetics for the formation of the DNA adducts in this experimental setup compared to what occurs in the biological environment.

**FIGURE 1:**
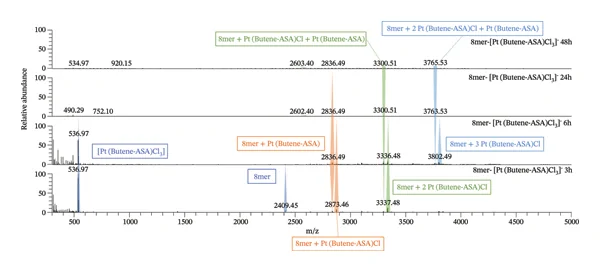
Time‐resolved deconvoluted HR‐ESI mass spectra for the incubation reaction of **1** with 8mer (3:1).

Compound **1** exhibited lower reactivity toward the model oligonucleotide compared to DDP (Figure 1, Table S6). The parent complex and the 8mer peak disappeared within 24 h, leading to oligonucleotide adducts. However, hydrolysis and degradation also play a role for this particular compound, as it exhibited the lowest stability in aqueous media among the compounds used in this study [10]. The formation of mono‐platinated ([8mer + Pt(Butene‐ASA)Cl] and [8mer + Pt(Butene‐ASA)]) and di‐platinated adducts ([8mer + 2 Pt(Butene‐ASA)Cl] and [8mer + Pt(Butene‐ASA)Cl + Pt(Butene‐ASA)]) was already observed after 3 h. Tri‐platinated species ([8mer + 3 Pt(Butene‐ASA)Cl] and [8mer + 2 Pt(Butene‐ASA)Cl + Pt(Butene‐ASA)]) became relevant after 6h of incubation, albeit all adducts appeared at low relative abundance.

Compound **1** has a labile chlorido ligand in the *trans* position to the olefin that is easily replaced by other molecules, such as solvent molecules or accessible nitrogen donors in the oligonucleotide. The olefinic ligand exerts a stronger *trans* effect than the *cis* chlorido ligands. Consequently, the two *cis* chlorido ligands are substantially more stable than the chlorido ligand *trans* to the olefin [34–37].

**SCHEME 2:**
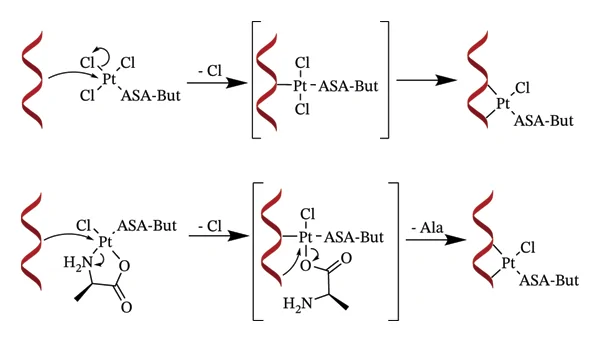
Proposed mechanism for the reaction of Compound **1** (above) and Compound **2** (below) with the oligonucleotide 8mer.

After the substitution of the first chlorido ligand, a second chlorido ligand can also be replaced to allow the formation of a more stable chelated oligonucleotide adduct (see Scheme 2, top). The species [8mer + Pt(Butene‐ASA)] could feature a tridentate‐coordinated oligonucleotide or, more likely, the di‐chelated species where a coordination vacancy is formed in the gas phase after loss of a loosely bound ligand, such as a solvent molecule.

**FIGURE 2:**
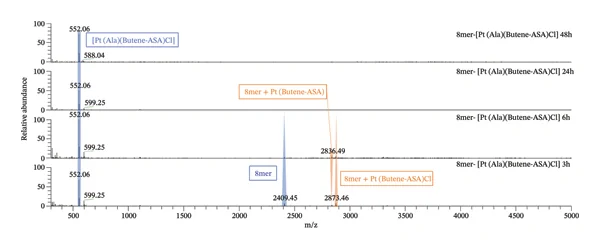
Time‐resolved deconvoluted HR‐ESI mass spectra for the incubation reaction of **2** with 8mer (3:1).

Compound **2** exhibited low reactivity toward the model oligonucleotide, with the molecular ion of the complex remaining the base peak throughout the entire experiment (Figure 2, Table S7). Mainly two platinum‐containing oligonucleotide adducts were detected, [8mer + Pt(Butene‐ASA)Cl] and [8mer + Pt(Butene‐ASA)], with limited abundance compared to the base peak. Further minor adducts are listed in Table S7. It is worth noting that none of the reported adducts contain the amino acid ligand, suggesting that after the L‐Ala chelate ring is opened, the amino acid is easily detached from the platinum center. Compound **1** showed distinctly different speciation depending on the incoming donor being N, as in oligonucleotides, or S, as in amino acids Met and Cys [11].

The *trans* effects of the ligands in Compound **2** can be ranked as follows: olefin > Cl^−^ > RNH_2_ ≈ R’COO^−^ [34–39].

In agreement with the predicted strong *trans* effect of the olefinic ligand [34, 35, 37], a nucleophilic attack of a nucleobase is accompanied by the opening of the amino acid chelating ring (Scheme 2, bottom). Subsequently, the *trans* effect of the chlorido ligand [34–36] promotes dissociation of the alanine ligand from the platinum center, allowing the oligonucleotide to coordinate in a bidentate manner. This process is favored by the lower affinity of platinum for oxygen donors compared to nitrogen donors, in agreement with HSAB principles. Also, bidentate coordination of the 8mer stabilizes the adduct through the chelate effect. The so‐obtained intermediate ([8mer + Pt(Butene‐ASA)Cl]) and the adduct generated in the next step ([8mer + Pt(Butene‐ASA)]) are identical to those observed for Compound **1**.

The comparison of the spectra obtained from the experiments with DDP, OxPt, **1**, and **2** clearly highlights the relative inertness of Compound **2** toward oligonucleotides compared to the other platinum complexes. Nonetheless, Compound **2** demonstrated cytotoxic activity comparable to DDP in several cancer cell lines [10] and prompted us to investigate the molecular interactions with a model peptide and protein.

#### 3.1.2. Angiotensin I

AT1 features two His residues in its sequence (see Table S1), which have been reported to coordinate to a number of different metal complexes previously [9, 18, 19, 40].

DDP showed only marginal interaction with AT1 (Figure S3, Table S8). Only after 24 h of incubation, a low‐abundant signal for [AT1 + Pt(NH_3_)_2_] was observed, in agreement with previous studies [18].

The incubation of OxPt with AT1 also led to low‐abundant adducts only Figure S4, Table S9), which were identified as [AT1 + Pt(Ox)(DACH)] and, later, [AT1 + Pt(DACH)].

Compound **1** formed a clearly detectable adduct upon incubation with AT1 corresponding to [AT1 + Pt(Butene‐ASA)Cl] (Figure S5, Table S10), as also previously observed [9].

**FIGURE 3:**
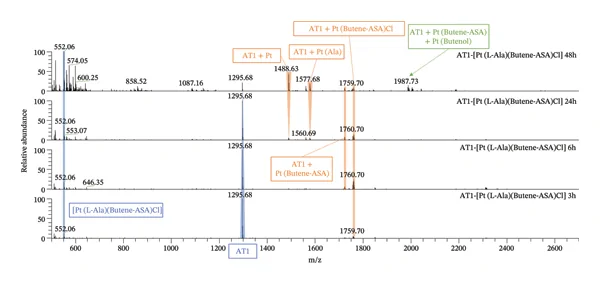
Time‐resolved deconvoluted HR‐ESI mass spectra for the incubation reaction of **2** with AT1 (3:1).

The incubation of Compound **2** with AT1 produced more adducts than those formed by the other three platinum complexes, including some di‐platinated species (Figure 3, Table S11). The adduct [AT1 + Pt(Butene‐ASA)Cl] formed quickly and remained present over the course of the experiment. The chelating alanine ligand has been lost in this adduct, in an analogous mechanism as described for the oligonucleotide above (Scheme 2). Over the course of the reaction, a number of low‐abundant di‐platinated species were detected, many of which contained the Ala ligand (see Table S11). After 48 h, an intense peak was identified as [AT1 + Pt], which has lost all of the original ligands on the platinum center. Despite the formation of many adducts, the intact complex as well as the unmodified peptide remained clearly detectable for 48 h.

#### 3.1.3. Cytochrome c

CytC used in this study was provided as ferricytochrome c (with Fe(III) in the heme group). If incubated at 37°C, even in the absence of a platinum complex, CytC underwent rapid oxidation and a cluster of signals corresponding to CytC + O appeared in the mass spectrum. This effect was drastically reduced when incubating the CytC at room temperature and protected from light. Data shown here represent these optimized conditions for CytC incubation experiments. We also observed a drastic drop in the signal intensity after 48 h; hence, we report only the results up until 24 h of incubation. Similar conditions were used within our group, investigating the reaction of DDP and Complex **2** with various model proteins, including CytC [41].

CytC offers a number of potential binding sites. On the basis of the HSAB theory, sulfur‐donor residues have the highest affinity for platinum. Such residues are present as two methionine groups, Met65 and Met80, together with two cysteines, Cys14 and Cys17, that anchor the heme group via the formation of thioethers. Met65 is the only sulfur‐containing residue that is readily solvent‐accessible, while the others are located in proximity to the heme group (Met80 coordinates Fe(III) in the native conformation). In addition, there are several nitrogen donor residues in the sequence of CytC, especially His18, His26, and His33, which are often recognized as potential binding sites for platinum complexes.

The incubation reaction of CytC with DDP has been extensively studied previously [15, 24, 41–44]. In our investigations, we observed a limited number of adducts with low intensities compared to the free protein (Figure S6, Table S12). The most relevant adducts were [CytC + Pt(NH_3_)_2_Cl_2_] and [CytC + Pt(NH_3_)_2_Cl] species. These results align well with the literature, particularly when a comparable ratio of platinum compound to protein was used [15, 43, 45].

A similar outcome was observed for the incubation reaction of CytC with OxPt (Figure S7, Table S13). In this case, the most relevant adduct was identified as [CytC + Pt(DACH)(Ox)], which slowly converted into [CytC + Pt(DACH)]. A di‐metalated adduct [CytC + 2 Pt(DACH)(Ox)] was also observed. Again, these results are consistent with similar investigations [15].

**FIGURE 4:**
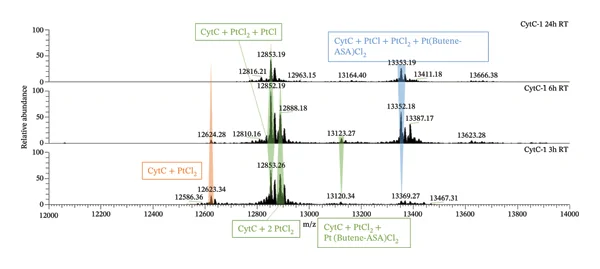
Time‐resolved deconvoluted HR‐ESI mass spectra for the incubation reaction of **1** with CytC at room temperature.

The rate of the reaction between CytC and Compound **1** is faster than that of any other platinum‐based anticancer agent, with complete consumption of CytC after 3 h of incubation (Figure 4, Table S14). Already at the first time point, several platinated species were observed. Only one mono‐platinated adduct was clearly observed, [CytC + PtCl_2_], in which one chloride ligand and the olefinic ligand have been replaced. This adduct was consumed over time and was barely detectable after 24 h of incubation. The region of di‐platinated species showed a number of high‐abundant signals from the first time point onward. We identified mainly two adducts, [CytC + 2 PtCl_2_] and [CytC + PtCl_2_ + PtCl], and their corresponding oxidation products, which represent the most abundant species throughout the whole experiment. Starting from 6 h of incubation, tri‐platinated species were gaining abundance, especially [CytC + 2 PtCl_2_ + Pt(ASA‐Butene)Cl_2_] and [CytC + PtCl + PtCl_2_ + Pt(ASA‐Butene)Cl_2_].

We observed the formation of a brown precipitate in the reaction vessel, likely a product of aggregation of platinated proteins.

**FIGURE 5:**
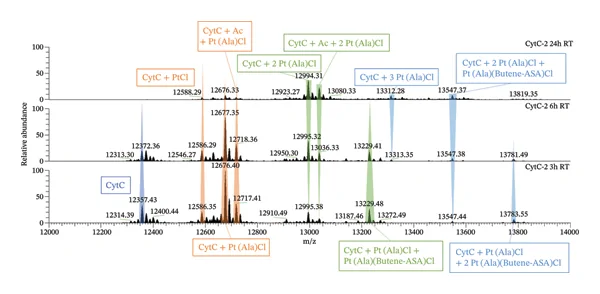
Time‐resolved deconvoluted HR‐ESI mass spectra for the incubation reaction of **2** with CytC at room temperature.

The reaction of Compound **2** with CytC exhibited slower kinetics compared to Compound **1** but demonstrated a distinctly enhanced reactivity relative to the platinum‐based anticancer drugs DDP and OxPt (Figure 5, Table S15). CytC was consumed after 24 h of incubation. After 3 h of incubation, the most abundant species were mono‐platinated adducts, particularly [CytC + Pt(Ala)(Butene‐ASA)Cl], [CytC + Pt(Ala)Cl], and [CytC + PtCl]. Most of these adducts retained the Ala ligand, while they lost the olefinic ligand. These adducts were consumed over time and almost vanished after 24 h. Several di‐platinated adducts were also detected after 3 h, such as [CytC + Pt(Ala)(Butene‐ASA)Cl + Pt(Ala)Cl] and [CytC + 2 Pt(Ala)Cl]. The former was progressively converted into the latter over time, as demonstrated by the high‐abundant signal for [CytC + 2 Pt(Ala)Cl] after 24 h of incubation. Low‐abundant tri‐metalated species are also observed starting from 3 h of incubation, such as [CytC + 2 Pt(Ala)(Butene‐ASA)Cl + Pt(Ala)Cl], [CytC + Pt(Ala)(Butene‐ASA)Cl + 2 Pt(Ala)Cl], and [CytC + 3 Pt(Ala)Cl].

**SCHEME 3:**
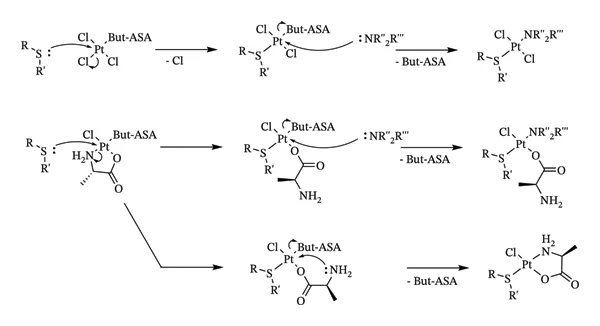
Reaction mechanism leading to the formation of mono‐platinated adducts for Compounds **1** (top) and **2** (bottom). A similar mechanism likely applies also for additional platinum units.

Based on the findings reported above and considering the *trans* effect and HSAB principles, the first step of the reaction of Zeise’s salt derivatives with CytC involved the exchange of the ligand in the *trans* position to the olefinic ligand with a thioether group of the protein. The *trans* effect exerted by the sulfur ligand then induced the loss of the olefin, as already observed for the reaction of several Zeise’s salt derivatives in the presence of S‐donor molecules [11]. The olefinic ligand was replaced by an N‐containing residue of a second amino acid of the protein or by back‐chelation of Ala, resulting in the [PtCl_2_] or the [Pt(Ala)Cl] adduct for Compounds **1** and **2**, respectively (Scheme 3).

The reactivity of Zeise’s salt derivatives toward CytC shares some similarities with non–platinum‐based anticancer agents, such as RAPTA‐C, rather than with canonical platinum‐based chemotherapeutics. In fact, DDP and OxPt incubated with CytC primarily produce mono‐platinated adducts, whereas the reactions of CytC with Zeise’s salt derivatives and RAPTA‐C result in multi‐metalated products, suggesting a preference for protein binding [44].

### 3.2. Binding Site Identification via MS/MS

In order to gain a more comprehensive picture of the various platinum adducts, HCD experiments were performed. Ions of interest were isolated in such a way that all isotopes of the respective envelope were included and subjected to increasing amounts of NCE to reach an optimum of fragment ions.

#### 3.2.1. Oligonucleotide

**FIGURE 6:**
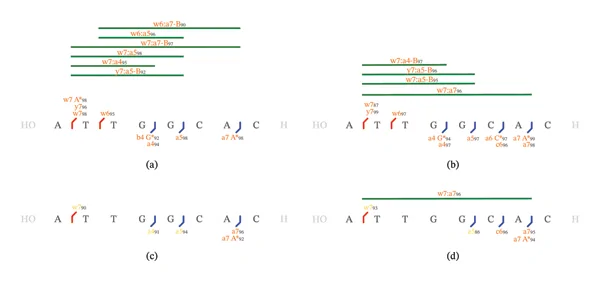
Fragmentation map of the peak (a) at m/z 526 ([8mer + Pt(NH_3_)_2_]^5−^), (b) at m/z 678 ([8mer + Pt(DACH) + H]^4−^), (c) at m/z 566 ([8mer + Pt(Butene‐ASA)]^5-^) for Compound **1**, and (d) Compound **2**. Platinated fragments are highlighted in orange, and platinated fragments detected applying a threshold of 0.5% in the setting of the Aom^2^S are highlighted in yellow.

The signal of the mono‐platinated adduct [8mer + Pt(NH_3_)_2_]^5−^ (m/z 526), detected during the incubation reaction of DDP with the 8mer, was dissociated by HCD with NCE increasing from 0 to 25 NCE units (fragmentation map in Figure 6a, Table S17).

Several fragments indicate the ‐GG‐ region as a binding site, such as [w6 + Pt(NH_3_)_1-2_ + 2 H]^3−^, [a5 + Pt(NH_3_) − H]^2−^, and [w6:a5 + Pt(NH_3_)]^−^, in good agreement with the data reported in the literature [3, 4, 13]. Noteworthy, alongside the abovementioned fragments, it was possible to observe [Pt(NH_3_) + g − 2 H]^−^ at m/z 360.02 that confirms the region ‐GG‐ as binding site, as also reported by Artner et al. [16].

**FIGURE 7:**
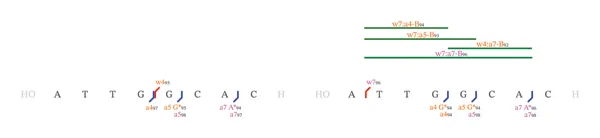
Fragmentation map of the peak at m/z 953 ([8mer + 2 Pt(NH_3_)_2_]^3−^) (left) and of the peak at m/z 756 ([8mer + 2 Pt(DACH) ‐ H]^4−^) (right). Mono‐platinated fragments are highlighted in orange, and di‐platinated fragments are highlighted in fuchsia.

We also attempted to identify the platination sites of the di‐platinated oligonucleotide. MS/MS spectra of the adduct [8mer + 2 Pt(NH_3_)_2_]^3−^ resulted in fragments [a4 + Pt(NH_3_) − H]^−^ and [a5 G∗ + Pt(NH_3_)_1-2_ − H]^2−^, confirming ‐GG‐ as the primary binding site (fragmentation map in Figure 7, Table S18). Considering the selectivity of DDP for purine nucleobases, it is expected that the second [Pt(NH_3_)_2_] moiety coordinates to one of the two adenines. Similar conclusions were also drawn from Artner et al. when analyzing the MS/MS of the same adduct [16].

OxPt has been reported to exhibit binding preferences similar to those of DDP for DNA nucleobases, interacting to a greater extent with purine bases than with pyrimidines, and showing the highest affinity for guanine [17, 32, 46–49]. Fragmentation of the adduct [8mer + Pt(Ox)(DACH) + 2H]^5−^ (m/z 560) produced only cleavage of the Pt‐oligonucleotide bond, even at low NCE values, resulting in peaks corresponding to the nonplatinated oligonucleotide and the parent anticancer drug (data not shown). These results suggest a weak interaction of the anticancer agent with the oligonucleotide, most likely a noncovalent adduct.

The fragmentation of the adduct [8mer + Pt(DACH) + H]^4−^ (m/z 678) produced several platinated species (fragmentation map in Figure 6b, Table S19). Several fragments, including [w6 + Pt(DACH) + 3 H]^2−^, [a4 G∗ + Pt(DACH) − H]^−^, [a4 + Pt(DACH) − 2 H]^2−^, and [a5 + Pt(DACH) − H]^2−^, indicate the ‐GG‐ section as the binding site, as expected. Two platinated fragments [Pt(DACH) + g − 2 H]^−^ and [Pt(DACH) + 2 g − H]^−^ at m/z = 608.16 and m/z = 457.11, respectively, further confirm this conclusion. The presence of the fragment [w7:a4‐G + Pt(DACH)]^−^ indicates a platination site within the sequence ‐TTf‐, suggesting the involvement of at least one thymine nucleobase. The formation of small amounts of bifunctional adducts involving both purine and pyrimidine bases has also been reported by Le Pla et al. [46]. Overall, the obtained fragmentation map is comparable to that observed for DDP, and the results are consistent with the literature [17, 32, 46–49].

We also investigated the coordination mode of the di‐platinated adduct [8mer + 2 Pt(DACH) ‐ H]^4−^ (m/z 754, fragmentation map in Figure 7, Table S20). Fragments [a4 + Pt(DACH) − 2 H]^2−^, [a5 G∗ + Pt(DACH)]^−^, [w4:a7‐A + Pt(DACH)]^−^, and [w7:a5‐G + Pt(DACH) + H]^−^ are all consistent with the platination of the ‐GG‐ region. Analogously to DDP, it is reasonable to assume that the second platination site may involve monofunctional modification of the adenine nucleobase, as supported by nearly all mono‐ and di‐platinated fragments observed. An exception is represented by the internal fragment [w7:a4‐G + Pt(DACH)]^−^. This mono‐platinated fragment containing the ‐TTf‐ sequence, already observed for the mono‐platinated adduct, indicates the involvement of at least one thymine nucleobase in platinum coordination. These findings may be rationalized by the ability of OxPt to also interact with pyrimidine nucleobases, albeit to a lesser extent [46].

An adduct formed by Compound **1**, [8mer + Pt(butene‐ASA)]^5−^ (m/z 566), was isolated and fragmented to obtain information about the coordination site of this platinum complex on the 8mer (fragmentation map in Figure 6c, Table S21). The data obtained from the MS/MS experiments showed that platinated fragments only appeared at NCE 15 and higher as low‐abundant signals, pointing to a less stable coordination of Complex **1** to the 8mer compared to DDP and OxPt. The limited number of platinated fragments detected did not allow a conclusive determination of the binding site, despite [w7 + Pt + 2H]^4−^, [a4 + Pt ‐ 2H]^2−^, and [a5 + Pt ‐ H]^2−^ pointing to the two adjacent guanines, as for DDP and OxPt. The same adduct ([8mer + Pt(butene‐ASA)]^5-^, m/z 566) was isolated and fragmented to obtain information about the coordination site for Compound **2** (fragmentation map in Figure 6d, Table S22). The results obtained were nearly identical to those observed for Compound **1**. Fragmentation of di‐platinated adducts in the case of Compounds **1** and **2** was not possible due to the low intensity of the corresponding signals. Taken together, these data indicate that all the complexes investigated in this study exhibit similar coordination behavior toward the model oligonucleotide, and that the differences between Zeise’s salt derivatives and DDP or OxPt are mainly attributed to differences in reaction kinetics.

#### 3.2.2. Angiotensin I

For AT1, platinum is expected to preferentially coordinate to the two histidine residues. Alternatively, coordination may occur at the N‐terminus, involving the amino group and the side‐chain carboxylate of the aspartate [9, 18, 19, 40].

In the case of the reaction between AT1 and DDP, adducts were too low in abundance to be isolated and fragmented.

**FIGURE 8:**
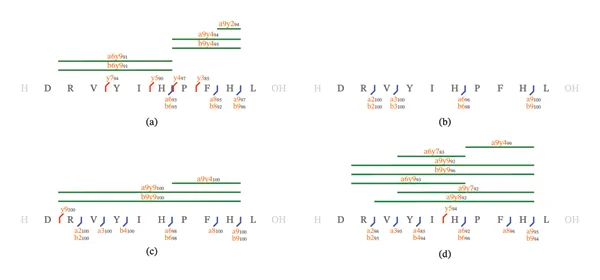
Fragmentation map of the peak at (a) m/z 535 ([AT1 + Pt(DACH) + H]^3+^), (b) m/z 898 ([AT1 + Pt(Butene‐ASA)Cl_2_ + 2 H]^2+^) from the reaction of AT1 with Compound **1**, (c) m/z 880 ([AT1 + Pt(Butene‐ASA)Cl + H]^2+^) from the reaction of AT1 with Compound **1**, and (d) m/z 880 ([AT1 + Pt(Butene‐ASA)Cl + H]^2+^) from the reaction of AT1 with Compound **2**.

The MS/MS spectra of the adduct [AT1 + Pt(DACH)(Ox) + 2H]^2+^ (m/z = 847) showed a progressive cleavage of the connection between OxPt and AT1 with increasing NCE, indicating a weak interaction between these two molecules, as previously observed for the 8mer (data not shown). A low‐abundance adduct at m/z 535 (*z* = 3), identified as [AT1 + Pt(DACH) + H]^3+^, was also subjected to HCD (Figure 8a, Table S23). In this case, fragmentation only happened above an NCE of 20, suggesting a more stable bidentate coordination.

**FIGURE 9:**
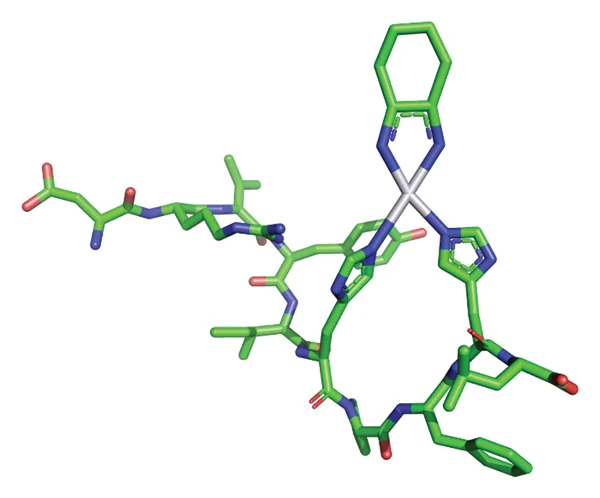
Proposed model for the coordination of platinum in the adduct [AT1 + PT(DACH)]^2+^. The [Pt(DACH)] unit was constructed onto AT1 based on PDB structure 1N9U, followed by geometry preoptimization using the UFF force field.

Fragments [y5 + Pt(DACH) ‐ H]^2+^, [a9 + Pt(DACH)]^3+^, and [b9 + Pt(DACH)]^3+^ restrict the binding sequence to the region between His6 and His9, excluding the coordination of platinum to the N‐terminus. The fragments [b6 + Pt(DACH) ‐ H]^2+^ and [y4 + Pt(DACH) ‐ H]^2+^ are complementary and point to one platinated His residue for each fragment. The same is true for the fragment [b6y9 + Pt(DACH) − H]^2+^ and the fragments [a9y4 + Pt(DACH) ‐ H]^2+^, [a9y4 + Pt ‐ 2 H]^+^, and [b9y4 + Pt(DACH) − H]^2+^. These data suggest that OxPt coordinates to AT1 via the two His residues. A further confirmation of this observation is the finding of a fragment where a [Pt(DACH)] moiety is coordinated to a single His (a9y2 or a6y5). The data collected confirm our expectations for OxPt to coordinate in a bis‐chelated fashion to the two His residues, as depicted in Figure 9.

Regarding the incubation reaction of Compound **1** with AT1, the peak of the monodentate adduct at m/z 898 ([AT1+Pt(Butene‐ASA)Cl_2_ + H]^2+^) was subjected to HCD (Figure 8b, Table S24). We observed the formation of several platinated fragments containing the His residues and the N‐terminus, such as [a9 + PtCl]^2+^, [a9 + Pt − H]^2+^, [b9 + PtCl]^2+^, [b9 + Pt − H]^2+^, [b6 + PtCl − H]^+^, and [b6 + PtCl − 2H]^+^. Additionally, we identified signals suggesting binding to the N‐terminus, like [a2 + Pt ‐ 2H]^+^, [b2 + Pt ‐ 2H]^+^, [a3 + PtCl − H]^+^, and [b3 + PtCl − H]^+^.

We also isolated and fragmented a bi‐dentate adduct from the same reaction, [AT1 + Pt(Butene‐ASA)Cl + H]^2+^ at m/z 880 (Figure 8c, Table S25). We identified several fragments indicating binding to His6 and/or His9, such as [a6 + PtCl]^2+^, [b6 + PtCl ‐ H]^+^, [a6/b6 + Pt − 2H]^+^, [a9/b9 + PtCl]^2+^, and [a9/b9 + Pt − H]^2+^. Several other fragments, such as [a2/b2 + Pt − 2H]^+^, [a3 + PtCl − H]^+^, [a3 + Pt − 2H]^+^, and [b4 + Pt − 2H]^+^, point also to the N‐terminus as binding site. These findings suggest that all three predicted platination sites can coordinate to platinum(II) ions from Compound **1,** and these results are in good agreement with the data previously reported by our group [9].

A mono‐dentate adduct ([AT1 + Pt(L‐Ala)(Butene‐ASA)Cl + 2H]^2+^ at m/z 925.38) from the incubation of AT1 with Compound **2** only resulted in the cleavage of the Pt moiety off the peptide upon increasing NCE, and hence, no binding site information could be gained for this precursor ion.

The bidentate adduct [AT1 + Pt(Butene‐ASA)Cl + H]^2+^ (m/z 880) from the reaction of AT1 with Compound **2** was fragmented as well (Figure 8d, Table S26). Several fragments ([a2/b2 + Pt − 2H]^+^, [a3 + Pt − 2H]^+^, and [a4/b4 + Pt − 2H]^+^) point to the coordination of platinum to the N‐terminus. Also, multiple internal fragments, particularly [a6y7 + Pt − 2H]^+^ and [a9y4 + Pt − 2H]^+^, as well as [a6 + PtCl]^2+^, [b6 + PtCl − H]^+^, [a6/b6 + Pt − 2H]^+^, [a9/b9 + PtCl]^2+^, [a9/b9 + Pt − H]^2+^, and [y5 + Pt − 2H]^+^, point to the His residues as coordination sites. These observations lead to the conclusion that the N‐terminus is likely involved, alongside the His residues, in the chelation of platinum for Compounds **1** and **2**, which differs from what was observed for OxPt. The steric demand of the butane‐ASA ligand might be responsible for this observation.

The di‐platinated adduct [AT1 + Pt(L‐Ala)(Butene‐ASA)Cl + Pt]^2+^ (m/z 1021) was also isolated and subjected to HCD. This specific adduct was selected because, among the detected di‐platinated species, it showed adequate intensity and was sufficiently resolved from other signals to allow reliable isolation for MS/MS fragmentation. The data obtained were not informative about the secondary binding site, analogously to what was observed for the fragmentation of the monodentate adduct [AT1 + Pt(L‐Ala)(Butene‐ASA)Cl + 2H]^2+^. We only observed the cleavage of the [Pt(L‐Ala)(Butene‐ASA)Cl] moiety from the adduct, as demonstrated by the formation of a cluster at m/z 745.82, recognized as the adduct [AT1 + Pt]^2+^.

Taken together, these data confirm the N‐terminus and the two His residues as distinct coordination sites on AT1 for Zeise’s salt derivatives, as previously observed [9, 18]. We observed in all HCD experiments of AT1 coordinated to Pt a high abundance of fragment ions cleaving the peptide bond between His6 and Pro7, which indicates a weakening of that particular peptide backbone bond in comparison to AT1 without Pt. This is further evidence for His6 as the primary binding site for all Pt compounds used in this study.

#### 3.2.3. Cytochrome c

Mainly bottom‐up approaches have been reported so far for the investigation of the metalation sites on CytC by DDP [23, 50]. Recently, an LC‐MS‐based top‐down approach was reported by our group [41]. However, under direct MS conditions, analysis of platinated CytC was not possible due to low signal intensity of the platinated adducts.

Previous studies report that DDP interacts with CytC primarily via Met65, which is solvent‐accessible [15, 21, 43, 45], and to a lesser degree, with Met80, competing with the iron of the heme group [41, 43]. Other publications suggested the histidine residues (His18, His26, and His33) as coordination sites [15, 43]. For Compounds **1** and **2**, we observed the loss of the olefinic ligands in the formed CytC adducts, which points to coordination through S donors (Met) based on previous studies [9, 11].

We observed a change in the color of the incubation reaction samples for CytC with Compounds **1** and **2**, while the samples of CytC with DDP, OxPt, and a CytC blank maintained their brilliant red coloration throughout the entire experiment (up to 48 h, Figure S9).

**FIGURE 10:**
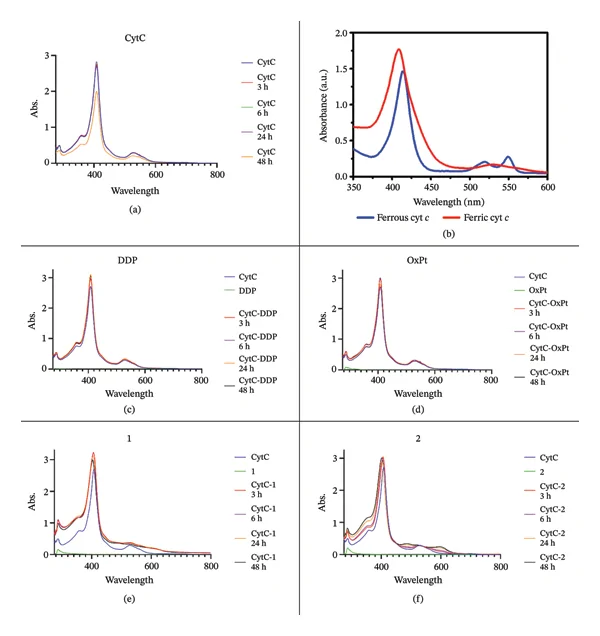
(a) Time‐resolved UV‐Vis spectra of the ferricytochrome c; (b) UV‐Vis spectra of ferric and ferrous CytC as reported in literature [51]; time‐resolved spectra of the incubation reaction of CytC with (c) DDP, (d) OxPt, (e) **1** and (f) **2**.

This observation prompted us to monitor the incubation reactions via UV‐Vis spectroscopy (Figure 10). The UV‐Vis spectrum of a CytC blank confirmed its ferric form as compared to Figure 10(b) [51] and showed no changes over time, except for a reduction in absorbance after 48 h (Figure 10(a)). Incubation of CytC with DDP and OxPt (Figure 10(c,d), respectively) resulted in no changes in the UV‐Vis spectra throughout the experiment compared to the control sample.

In the case of the incubation reaction of CytC with Compounds **1** and **2**, some changes were observed. Upon interaction with Compound **1**, the CytC spectrum showed an initial change and then remained constant after 3h. Compound **2** resulted in alterations throughout the 48 h of observation. In both cases, a hypsochromic shift of the Soret band from 408 nm to approximately 400 nm was detected. The Q bands at 530 nm and 555 nm also underwent a blue shift to roughly 485 nm and 520 nm, respectively. Also, the formation of a new charge‐transfer band is observed at around 600 nm. All these findings are consistent with the transition from a low‐spin (LS) state for the iron(III) in CytC to a high‐spin (HS) state [52]. These electronic transitions may be explained by the cleavage of the Met80–Fe(III) coordination bond and the exchange of the high‐field methionine ligand with a low‐field ligand, such as a water molecule, as was observed previously by Jiang et al. in the case of the interaction of CytC with trans‐diaminedichloridoplatinum(II) (transplatin) [52, 53]. To confirm this hypothesis, the band at 695 nm, corresponding to the Fe(III)–Met80 interaction [54], was monitored. It was observed that this band remained the same upon binding of DDP and OxPt to CytC, but disappeared after incubation with Zeise’s salt derivatives, similar to the incubation of CytC with 10 equivalents of transplatin (Figure S10) [15, 21, 38, 45, 54].

### 3.3. Limitations of the Study

In this study, we examined the reactivity of Zeise’s salt derivatives with three model biomolecules representing DNA, peptides, and proteins. The observed differences in reactivity, particularly for Compound **2**, suggest a contrast in the molecular targets and mode of action for Zeise’s salt derivatives compared to approved platinum drugs. However, the actual molecular target for these complexes is yet to be identified. Moreover, the present work evaluates reactivity with isolated models. A competitive assay including protein, peptide, and oligonucleotide models could further confirm whether this class of compounds shows a preference for one model over the other; however, this is nontrivial from an analytical point of view, as the polarity of ions in the gas phase is different for oligonucleotides and peptides.

It is also important to note that the experiments were conducted under simplified aqueous conditions because of the instrumental limitations of ESI‐MS. For example, high chloride concentrations, which strongly influence speciation of DDP and OxPt, are incompatible with MS analysis. Consequently, the conclusions drawn here do not capture the full set of transformations that these compounds may undergo in vivo.

Regarding the binding sites of Compounds **1** and **2** on the oligonucleotide, MS/MS data suggest behavior similar to that of DDP and OxPt. However, these conclusions should be interpreted with caution given the low abundance and limited number of platinated adducts detected.

The half‐life of Compound **1** in aqueous solution is approximately 70 h [7]. Its limited stability is more relevant at later time points (48 h), when about one‐quarter of the original complex has likely degraded. Nonetheless, no adducts derived from potential degradation products were detected with any of the models. The impact of aqueous instability can therefore be considered negligible at 3, 6, and 24 h. In particular, for the incubation of Compound **1** with CytC, the reaction kinetics are much faster than the degradation of the complex, so degradation effects can be disregarded.

## 4. Conclusion

In the present study, the reactivity of Zeise’s salt derivatives (Compounds **1** and **2**) toward different model biomolecules was investigated and compared to two reference anticancer agents, DDP and OxPt. At first, the ability of these substances to bind to DNA was studied using a model oligonucleotide (8mer) [16]. Compound **2** showed a distinctly lower reactivity toward the oligonucleotide than the other three compounds. Given the comparable in vitro cytotoxicity of DDP and Compound **2** against different cell lines [10], these results suggest a different molecular target for Compound **2**. Based on the results obtained from the top‐down investigation, all the complexes in this study share a similar mode of coordination to the oligonucleotide; however, the reaction kinetics of the four platinum compounds with DNA differ significantly.

We also investigated the reactivity of the Zeise’s salt derivatives **1** and **2** toward a peptide and a protein model, AT1 and CytC, respectively. Compound **1** exhibited the highest reactivity toward CytC, while Compound **2** showed enhanced reactivity toward CytC compared to the approved platinum‐based anticancer drugs DDP and OxPt, and the highest reactivity toward AT1. These results further support the hypothesis that the mode of action of Zeise’s salt derivatives should be investigated in terms of their interactions with peptides and proteins, rather than relying on DNA affinity, as their behavior toward CytC more closely resembles that of the ruthenium‐based RAPTA‐C than that of DDP or OxPt [44]. While OxPt was observed to coordinate to AT1 via the two His residues, the coordination of Zeise’s salt derivatives also involved the N‐terminus of AT1 (Asp1), alongside His6 and His9 residues. The Zeise’s salt derivatives **1** and **2** probably saturated the available thioether groups on CytC, rather than coordinating to the His residues, as reported for DDP [15, 43].

A possible explanation for the higher reactivity of Zeise’s salt derivatives toward CytC may lie in their broader adduct geometry options. Depending on the nature of the entering ligand, this class of compounds can form chelated adducts with bite angles of either 90° or 180°. The presence of the olefinic ligand serves as an indicator of coordination to S‐donor residues, based on the results of this and other studies conducted by our group [9, 11]. These findings not only enhance our understanding of the distinct reactivity profiles of Zeise’s salt derivatives compared to traditional platinum‐based anticancer agents but also open exciting avenues for further research into their interactions with peptides and proteins, potentially unveiling novel therapeutic strategies beyond targeting DNA.

## Funding

This research was funded by the Austrian Science Fund, DOI: 10.55776/P37034.

Open Access funding provided by Universitat Innsbruck.

## Conflicts of Interest

The authors declare no conflicts of interest.

## Supporting Information

Additional supporting information can be found online in the Supporting Information section.

## Supporting information


**Supporting Information 1** The supplementary material includes molecular formulas and sequence information for the 8mer, AT1, and CytC; lists of species detected during the MS experiments; time‐resolved deconvoluted HR‐ESI mass spectra for the reactions involving 8mer, AT1, and CytC; examples of isotopic distributions; Apm^2^S and Aom^2^S search parameters; MS/MS data for selected 8mer and AT1 adducts; and UV–Vis experiments on CytC.

## Data Availability

The data supporting this article have been included as part of the supporting information (SI). Additional data that support the findings of this study are available from the corresponding author upon reasonable request. No cell lines or other biological resources requiring Research Resource Identifiers (RRIDs) were used in the present study. Any mention of cytotoxicity data refers exclusively to previously published results and is included only to provide context for the investigated platinum(II) complexes.
