# Supplementary material for: All Platinum(II) Ions Are Equal, but Some React Differently: Insights Into the Biomolecular Reactivity of Platinum(II)‐Based Anticancer Agents
Source: Bioinorg Chem Appl. 2026 Aug 20;2026:9865583. doi: 10.1155/bca/9865583 (PMC13491152; doi:10.1155/bca/9865583)
Supplement: Supplementary file 1 [file BCA-2026-9865583-s001.pdf]

# Supporting information: All Platinum(II) Ions Are Equal, but Some React Differently: Insights into the Biomolecular Reactivity of Platinum(II)-Based Anticancer Agents

Andrea Cucchiaro[1], Monika Cziferszky[1]*

monika.cziferszky@uibk.ac.at

[1] *Institute of Pharmacy, Pharmaceutical Chemistry,* ***Center for Molecular Biosciences Innsbruck (CMBI),*** *University of Innsbruck, Innrain 80/82, A-6020 Innsbruck, Austria*

## Contents

MS data ........ 2

8mer ........ 2

AT1 ........ 4

CytC ........ 5

Stacked spectra ........ 7

8mer ........ 7

AT1 ........ 8

MS/MS ........ 10

8mer ........ 11

UV experiments ........ 17

References: ........ 18

## MS data

Table S1 Molecular formula for each of the model biomolecules included in this study. (*) = Heme group.

| Model | Sequence | Molecular Formula |
|---|---|---|
| **8mer$^{7-}$** | ATTGGCAC | $C_{78}H_{92}N_{30}O_{46}P_7$ |
| **AT1** | DRVYIHPFHL | $C_{62}H_{89}N_{17}O_{14}$ |
| **CytC$^{+}$** | AcGDVEKGKKIFVQKC(*)AQC(*)HTVEKGGKHKTGPNLHGLF GRKTGQAPGFTYTDANKNKGITWKEETLMEYLENPKKYIPGTK MIFAGIKKKTEREDLIAYLKKATNEE | $C_{560}H_{874}N_{148}O_{156}S_4Fe_1$ |

Table S2 Molecular formula for each of the ligands considered in this study

| Ligand | Molecular Formula |
|---|---|
| **DACH** | $C_6H_{14}N_2$ |
| **Ox$^{2-}$** | $C_2O_4$ |
| **ASA-Butene** | $C_{13}H_{14}O_4$ |
| **SA-Butene** | $C_{11}H_{12}O_3$ |
| **Ala$^{-}$** | $C_3H_6NO_2$ |

### 8mer

Table S3 List of detected species during the incubation of 8mer (negative mode).

| Species | $m/z_{exp}$ | $m/z_{calc}$ | Error [ppm] | Intensity |
|---|---|---|---|---|
| $[8mer + H]^{6-}$ | 400.4000 | 400.4009 | -2.2 | 8.22E+6 |
| $[8mer + 2 H]^{5-}$ | 480.6814 | 480.6826 | -2.5 | 8.18E+6 |
| $[8mer + 3 H]^{4-}$ | 601.1040 | 601.1050 | -1.7 | 2.98E+6 |
| $[8mer + 4 H]^{3-}$ | 801.8075 | 801.8092 | -2.1 | 1.43E+6 |
| $[8mer + 5 H]^{2-}$ | 1203.7162 | 1203.7187 | -2.1 | 1.30E+5 |
| $[8mer - a]^{6-}$ | 377.8907 | 377.8919 | -3.2 | 2.67E+5 |
| $[8mer - a + H]^{5-}$ | 453.6706 | 453.6717 | -2.4 | 2.29E+6 |
| $[8mer - t]^{6-}$ | 379.3927 | 379.3938 | -2.9 | 1.29E+5 |
| $[8mer - t + H]^{5-}$ | 455.4728 | 455.474 | -2.6 | 3.84E+5 |

Table S4 List of detected species during the incubation reaction between 8mer and DDP (negative mode). Mono-platinated adducts are highlighted in orange, di-platinated in green and tri-platinated in light blue.

| Species | $m/z_{exp}$ | $m/z_{calc}$ | Error [ppm] |
|---|---|---|---|
| $[8mer + Pt(NH_3)_2]^{5-}$ | 526.2826 | 526.2834 | -1.5 |
| $[8mer + 2Pt(NH_3)_2Cl]^{5-}$ | 586.2734 | 586.2742 | -1.4 |
| $[8mer + Pt(NH_3)_2Cl + Pt(NH_3)_2]^{4-}$ | 723.8495 | 723.8505 | -1.4 |
| $[8mer + 2Pt(NH_3)_2]^{3-}$ | 953.4762 | 953.4778 | -1.7 |
| $[8mer + 3Pt(NH_3)_2 - 2H]^{3-}$ | 1029.1436 | 1029.1450 | -1.5 |
| $[8mer + 3Pt(NH_3)_2Cl]^{4-}$ | 798.8378 | 798.839 | -1.5 |
| $[8mer + 2Pt(NH_3)_2Cl + Pt(NH_3)_2]^{3-}$ | 1053.461 | 1053.463 | -2.0 |
| $[8mer + Pt(NH_3)_2Cl + 2Pt(NH_3)_2 - H]^{3-}$ | 1041.1353 | 1041.1369 | -1.8 |
| $[8mer + 2Pt(NH_3)_2 + Pt(H_2O)(NH_3)_2 - 2H]^{3-}$ | 1035.1469 | 1035.1485 | -1.6 |
| $[8mer + Pt(NH_3)_2Cl + Pt(H_2O)(NH_3)_2 + Pt(NH_3)_2 - H]^{3-}$ | 1047.139 | 1047.141 | -1.6 |

Table S5 List of detected species during the incubation reaction between 8mer and OxPt (negative mode). Mono-platinated adducts are highlighted in orange, di-platinated in green and tri-platinated in light blue.

| Species | $m/z_{exp}$ | $m/z_{calc}$ | Error [ppm] |
|---|---|---|---|
| $[8mer + 2 H]^{5-}$ | 480.6817 | 480.6826 | -1.9 |
| $[Pt(Ox)(DACH) - H]^{-}$ | 396.0523 | 396.0529 | -1.5 |
| $[8mer + Pt(Ox)(DACH) + 2H]^{5-}$ | 560.2942 | 560.2950 | -1.4 |
| $[8mer + Pt(DACH)]^{5-}$ | 542.2950 | 542.2960 | -1.8 |
| $[8mer + 2 Pt(Ox)(DACH) + 2H]^{5-}$ | 639.7060 | 639.7070 | -1.6 |
| $[8mer + 2 Pt(DACH)]^{3-}$ | 1006.8512 | 1006.8529 | -1.7 |
| $[8mer + Pt(DACH) + Pt(Ox)(DACH)]^{5-}$ | 621.7069 | 621.7079 | -1.6 |
| $[8mer + 3Pt(Ox)(DACH) + 2H]^{5-}$ | 719.1179 | 719.1189 | -1.4 |
| $[8mer + Pt(DACH) + 2Pt(Ox)(DACH)]^{5-}$ | 701.1188 | 701.1198 | -1.4 |

Table S6 List of detected species during the incubation reaction between 8mer and **1** (negative mode). Mono-platinated adducts are highlighted in orange, di-platinated in green and tri-platinated in light blue.

| Species | $m/z_{exp}$ | $m/z_{calc}$ | Error [ppm] |
|---|---|---|---|
| $[Pt(butene\text{-}ASA)Cl_3]^{-}$ | 533.9608 | 533.9612 | -0.7 |
| $[PtCl_3]^{-}$ | 299.8714 | 299.8719 | -1.7 |
| $[8mer + 2 H]^{5-}$ | 480.6817 | 480.6826 | -1.9 |
| $[8mer + Pt(butene\text{-}ASA)Cl + H]^{5-}$ | 573.4853 | 573.4859 | -1.0 |
| $[8mer + Pt(butene\text{-}ASA)]^{5-}$ | 566.2900 | 566.2907 | -1.2 |
| $[8mer + 2 Pt(butene\text{-}ASA)Cl]^{5-}$ | 666.2882 | 666.2888 | -0.9 |
| $[8mer + Pt(butene\text{-}ASA)Cl + Pt(butene\text{-}ASA) - H]^{5-}$ | 658.8930 | 658.8935 | -0.8 |
| $[8mer + Pt(butene\text{-}ASA)Cl + Pt - H]^{5-}$ | 612.0748 | 612.0756 | -1.3 |
| $[8mer + Pt(butene\text{-}ASA)Cl + PtCl - H]^{6-}$ | 516.0571 | 516.0579 | -1.6 |

| | | | |
|---|---|---|---|
| [8mer + 3 Pt(butene-ASA)Cl - H]$^{5-}$ | 759.0908 | 759.0917 | -1.2 |
| [8mer + 2 Pt(butene-ASA)Cl + Pt(butene-ASA)Cl$_2$]$^{5-}$ | 766.2858 | 766.2869 | -1.4 |
| [8mer + 2 Pt(butene-ASA)Cl + Pt(butene-ASA) - 2H]$^{5-}$ | 751.6957 | 751.6964 | -0.9 |
| [8mer + 2 Pt(butene-ASA)Cl + PtCl - H]$^{5-}$ | 712.2730 | 712.2738 | -1.1 |

Table S7 List of detected species during the incubation reaction between 8mer and **2** (negative mode). Mono-platinated adducts are highlighted in orange and di-platinated in green.

| Species | $m/z_{exp}$ | $m/z_{calc}$ | Error [ppm] |
|---|---|---|---|
| [Pt(Ala)(butene-ASA)Cl - H]$^{-}$ | 551.0552 | 551.0556 | -0.7 |
| [Pt(Ala)Cl - H]$^{-}$ | 316.9657 | 316.9662 | -1.6 |
| [Pt(Ala)(butene-SA)Cl - H]$^{-}$ | 509.0443 | 509.0450 | -1.4 |
| [Pt(Ala)(butene-ASA)Cl + Cl]$^{-}$ | 587.0318 | 587.0323 | -0.9 |
| [8mer + 2 H]$^{5-}$ | 480.6818 | 480.6826 | -1.7 |
| [8mer + Pt(Ala)(butene-ASA)Cl + 4H]$^{3-}$ | 986.1628 | 986.1639 | -1.1 |
| [8mer + Pt(butene-ASA)Cl + 2H]$^{4-}$ | 717.1084 | 717.1091 | -1.0 |
| [8mer + Pt(butene-ASA) + H]$^{4-}$ | 708.1145 | 708.1152 | -1.0 |
| [8mer + 2Pt(butene-ASA) - H]$^{4-}$ | 814.8739 | 814.8747 | -1.0 |
| [8mer + 2Pt(butene-ASA)Cl + H]$^{4-}$ | 833.1119 | 833.1128 | -1.1 |
| [8mer + Pt(butene-ASA)Cl + Pt(butene-ASA)]$^{4-}$ | 824.1181 | 824.1189 | -1.0 |

## AT1

Table S8 List of detected species during the incubation reaction between AT1 and DDP (positive mode). Mono-platinated adducts are highlighted in orange.

| Species | $m/z_{exp}$ | $m/z_{calc}$ | Error [ppm] |
|---|---|---|---|
| [AT1 + 3 H]$^{3+}$ | 432.9001 | 432.8998 | 0.7 |
| [AT1 + Pt(NH$_3$)$_2$ + H]$^{3+}$ | 508.9014 | 508.9011 | 0.6 |

Table S9 List of detected species during the incubation reaction between AT1 and OxPt (positive mode). Mono-platinated adducts are highlighted in orange.

| Species | $m/z_{exp}$ | $m/z_{calc}$ | Error [ppm] |
|---|---|---|---|
| [OxPt + H]$^{+}$ | 398.0677 | 398.0675 | 0.5 |
| [AT1 + 2 H]$^{2+}$ | 648.8464 | 648.8460 | 0.6 |
| [AT1 + Pt(DACH)(Ox) + 2 H]$^{2+}$ | 847.8773 | 847.8771 | 0.2 |
| [AT1 + Pt(DACH) + H]$^{3+}$ | 535.5892 | 535.5887 | 0.9 |

Table S10 List of detected species during the incubation reaction between AT1 and **1** (positive mode). Mono-platinated adducts are highlighted in orange.

| Species | $m/z_{exp}$ | $m/z_{calc}$ | Error [ppm] |
|---|---|---|---|
| [AT1 + 3 H]$^{3+}$ | 432.9009 | 432.8998 | 2.5 |
| [AT1 + Pt(Butene-ASA)Cl + H]$^{2+}$ | 880.8569 | 880.8541 | 3.2 |
| [AT1 + Pt(Butene-ASA)Cl$_2$ + 2 H]$^{2+}$ | 898.8453 | 898.8421 | 3.5 |

| | | | |
|---|---|---|---|
| [AT1 + Pt(Butene-ASA)]$^{2+}$ | 862.8702 | 862.8662 | 4.6 |
| [AT1 + Pt(Butene-ASA) + $H_2O$]$^{2+}$ | 871.3729 | 871.3707 | 2.5 |
| [AT1 + Pt]$^{2+}$ | 745.3232 | 745.3207 | 3.6 |
| [AT1 + Pt(Butenol)]$^{2+}$ | 781.8530 | 781.8503 | 3.5 |

Table S11 List of detected species during the incubation reaction between AT1 and **2** (positive mode). Mono-platinated adducts are highlighted in orange and di-platinated in green.

| Species | m/$z_{exp}$ | m/$z_{calc}$ | Error [ppm] |
|---|---|---|---|
| [Pt(L-Ala)(Butene-ASA)Cl + H]$^{+}$ | 553.0715 | 553.0701 | 2.5 |
| [AT1+Pt(L-Ala)(Butene-ASA)Cl + 2 H]$^{2+}$ | 925.3797 | 925.3780 | 1.8 |
| [AT1+Pt(Butene-ASA)Cl + H]$^{2+}$ | 880.8556 | 880.8541 | 1.7 |
| [AT1 + Pt(Butene-ASA)]$^{2+}$ | 862.8686 | 862.8662 | 2.8 |
| [AT1 + Pt(Butene-ASA) + $H_2O$]$^{2+}$ | 871.8735 | 871.8715 | 2.3 |
| [AT1 + Pt]$^{2+}$ | 745.8235 | 745.8215 | 2.7 |
| [AT1 + Pt(L-Ala) + H]$^{2+}$ | 790.3476 | 790.3454 | 2.8 |
| [AT1 + Pt(Butenol)]$^{2+}$ | 781.8520 | 781.8503 | 2.2 |
| [AT1 + Pt(L-Ala)(Butene-ASA)Cl + Pt(Butene-ASA)Cl + H]$^{2+}$ | 1157.3866 | 1157.3852 | 1.2 |
| [AT1 + Pt(L-Ala)(Butene-ASA)Cl + Pt]$^{2+}$ | 1021.8540 | 1021.8523 | 1.7 |
| [AT1 + Pt(Butene-ASA)Cl + Pt(Butene-ASA) - H]$^{2+}$ | 1094.3746 | 1094.3731 | 1.4 |
| [AT1 + Pt(Butene-ASA) + Pt(Butenol) - 2 H]$^{2+}$ | 944.8702 | 944.8685 | 1.8 |
| [AT1 + Pt(L-Ala)(Butene-ASA) + Pt - H]$^{2+}$ | 1003.8665 | 1003.8645 | 2.0 |

## CytC

Table S12 List of detected species during the incubation reaction between CytC and DDP at RT (positive mode). Mono-platinated adducts are highlighted in orange.

| Species | m/$z_{exp}$ | m/$z_{calc}$ | Error [ppm] |
|---|---|---|---|
| [CytC + 14 H]$^{15+}$ | 824.8976 | 824.8963 | 1.6 |
| [CytC + O + 14 H]$^{15+}$ | 825.9640 | 825.9625 | 1.8 |
| [CytC + $Pt(NH_3)_2Cl_2$ + 14 H]$^{15+}$ | 844.8944 | 844.8931 | 1.5 |
| [CytC + $Pt(NH_3)_2Cl_2$ + O + 14 H]$^{15+}$ | 845.9607 | 845.9595 | 1.4 |
| [CytC + $Pt(NH_3)_2Cl$ + 13 H]$^{15+}$ | 842.4287 | 842.4282 | 0.6 |
| [CytC + $Pt(NH_3)_2Cl$ + O + 13 H]$^{15+}$ | 843.5619 | 842.5616 | 0.4 |

Table S13 List of detected species during the incubation reaction between CytC and OxPt at RT (positive mode). Mono-platinated adducts are highlighted in orange and di-platinated in green.

| Species | m/$z_{exp}$ | m/$z_{calc}$ | Error [ppm] |
|---|---|---|---|
| [$Pt(C_6H_{14}N_2)(C_2O_4)$ + H]$^{+}$ | 398.0687 | 398.0675 | 3.0 |
| [CytC + 14H]$^{15+}$ | 824.8978 | 824.8963 | 1.8 |
| [CytC + O + 14H]$^{15+}$ | 825.9641 | 825.9625 | 1.9 |
| [CytC + OxPt + 14H]$^{15+}$ | 851.3692 | 851.3668 | 2.8 |
| [CytC + OxPt + O + 14H]$^{15+}$ | 852.4349 | 851.4332 | 2.0 |

| | | | |
|---|---|---|---|
| [CytC + Pt(DACH) + 12H]$^{15+}$ | 845.3687 | 845.3673 | 1.7 |
| [CytC + Pt(DACH) + O + 12H]$^{15+}$ | 846.4353 | 846.4337 | 1.9 |
| [CytC + 2 OxPt + 14 H]$^{15+}$ | 877.8398 | 877.8377 | 2.4 |

Table S14 List of detected species during the incubation reaction between CytC and **1** at RT (positive mode). Mono-platinated adducts are highlighted in orange, di-platinated in green and tri-platinated in light blue.

| Species | $m/z_{exp}$ | $m/z_{calc}$ | Error [ppm] |
|---|---|---|---|
| [CytC + $PtCl_2$ + 12H]$^{13+}$ | 972.1050 | 972.1025 | 2.6 |
| [CytC + 2 $PtCl_2$ + 12H]$^{13+}$ | 992.4826 | 992.4793 | 3.3 |
| [CytC + 2 $PtCl_2$ + O + 12H]$^{13+}$ | 993.7128 | 993.7094 | 3.4 |
| [CytC + $PtCl_2$ + PtCl + 11H]$^{13+}$ | 989.7148 | 989.7119 | 2.9 |
| [CytC + $PtCl_2$ + PtCl + O + 11H]$^{13+}$ | 990.9448 | 990.9420 | 2.8 |
| [CytC + $PtCl_2$ + Pt(Butene-ASA)$Cl_2$ + 12H]$^{13+}$ | 1010.4900 | 1010.4861 | 3.9 |
| [CytC + PtCl + $PtCl_2$ + Pt(Butene-ASA)$Cl_2$ + 11H]$^{13+}$ | 1028.1755 | 1028.1726 | 2.8 |
| [CytC + PtCl + $PtCl_2$ + Pt(Butene-ASA)$Cl_2$ + 11H + O]$^{13+}$ | 1029.4824 | 1029.4803 | 2.0 |

Table S15 List of detected species during the incubation reaction between CytC and **2** RT (positive mode). Mono-platinated adducts are highlighted in orange, di-platinated in green and tri-platinated in light blue.

| Species | $m/z_{exp}$ | $m/z_{calc}$ | Error [ppm] |
|---|---|---|---|
| [Pt(Ala)(Butene-ASA)Cl + H]$^{+}$ | 553.0720 | 553.0701 | 3.4 |
| [CytC + 14H]$^{15+}$ | 824.8985 | 824.8963 | 2.7 |
| [CytC + PtCl + 13H]$^{15+}$ | 840.1607 | 840.1578 | 3.5 |
| [CytC + PtCl + O + 13H]$^{15+}$ | 841.2264 | 841.2242 | 2.6 |
| [CytC + Pt(Ala)Cl + 14H]$^{15+}$ | 846.0970 | 846.0944 | 3.1 |
| [CytC + Pt(Ala)Cl + O + 14H]$^{15+}$ | 847.1633 | 847.1608 | 3.0 |
| [CytC + Ac + Pt(Ala)Cl + 15H]$^{15+}$ | 848.9646 | 848.9623 | 2.7 |
| [CytC + Ac + Pt(Ala)Cl + O + 15H]$^{15+}$ | 850.0310 | 850.0284 | 3.1 |
| [CytC + 2 Pt(Ala)Cl + 14H]$^{15+}$ | 867.2947 | 867.2924 | 2.7 |
| [CytC + 2 Pt(Ala)Cl + O + 14H]$^{15+}$ | 868.4281 | 868.4259 | 2.5 |
| [CytC + Ac + Pt(Ala)Cl + Pt(Ala)Cl + 14H]$^{15+}$ | 870.0952 | 870.0929 | 2.6 |
| [CytC + Ac + Pt(Ala)Cl + Pt(Ala)Cl + O + 14H]$^{15+}$ | 871.1619 | 871.1593 | 3.0 |
| [CytC + Pt(Ala)Cl + Pt(Ala)(Butene-ASA)Cl + 14H]$^{15+}$ | 882.9682 | 882.9654 | 3.2 |
| [CytC + 3 Pt(Ala)Cl + 13H]$^{15+}$ | 888.4919 | 888.4901 | 2.0 |
| [CytC + 2 Pt(Ala)Cl + Pt(Ala)(Butene-ASA)Cl + 13H]$^{15+}$ | 904.0991 | 904.0961 | 3.3 |
| [CytC + Pt(Ala)Cl + 2 Pt(Ala)(Butene-ASA)Cl + 13H]$^{15+}$ | 919.7052 | 919.7018 | 3.7 |

# Stacked spectra

## 8mer

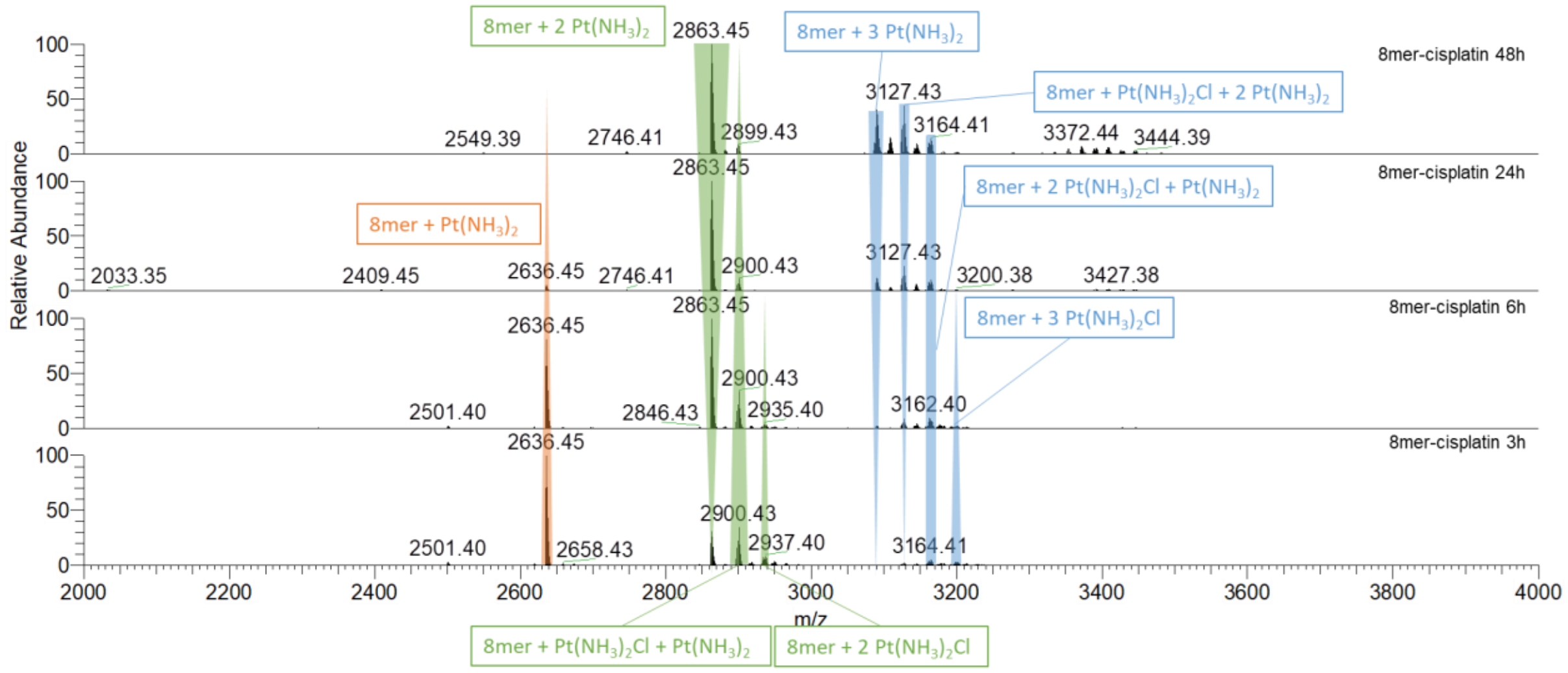


Figure S1 Time-resolved deconvoluted HR-ESI mass spectra for the incubation reaction of DDP with 8mer (3:1)

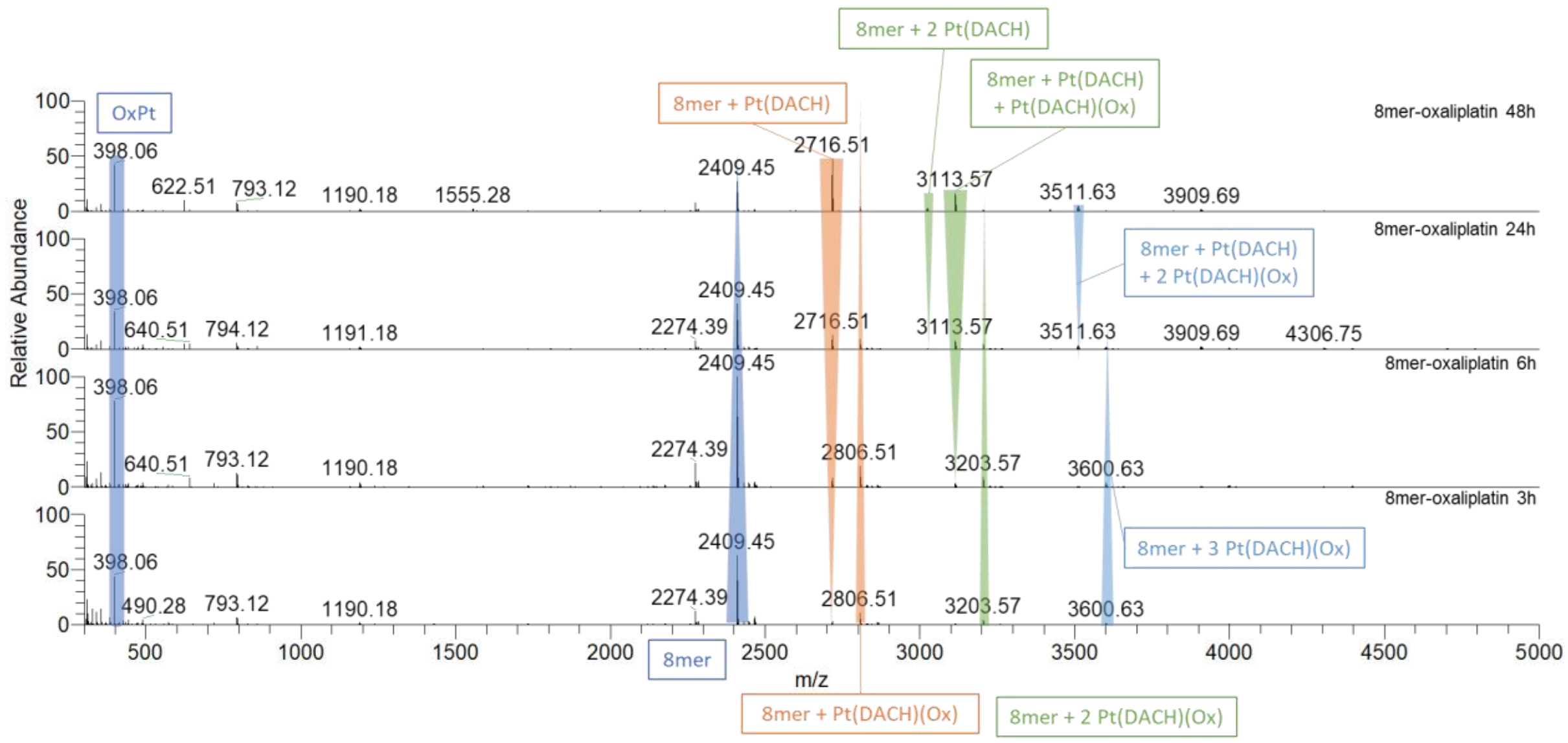


Figure S2 Time-resolved deconvoluted HR-ESI mass spectra for the incubation reaction of OxPt with 8mer (3:1)

## AT1

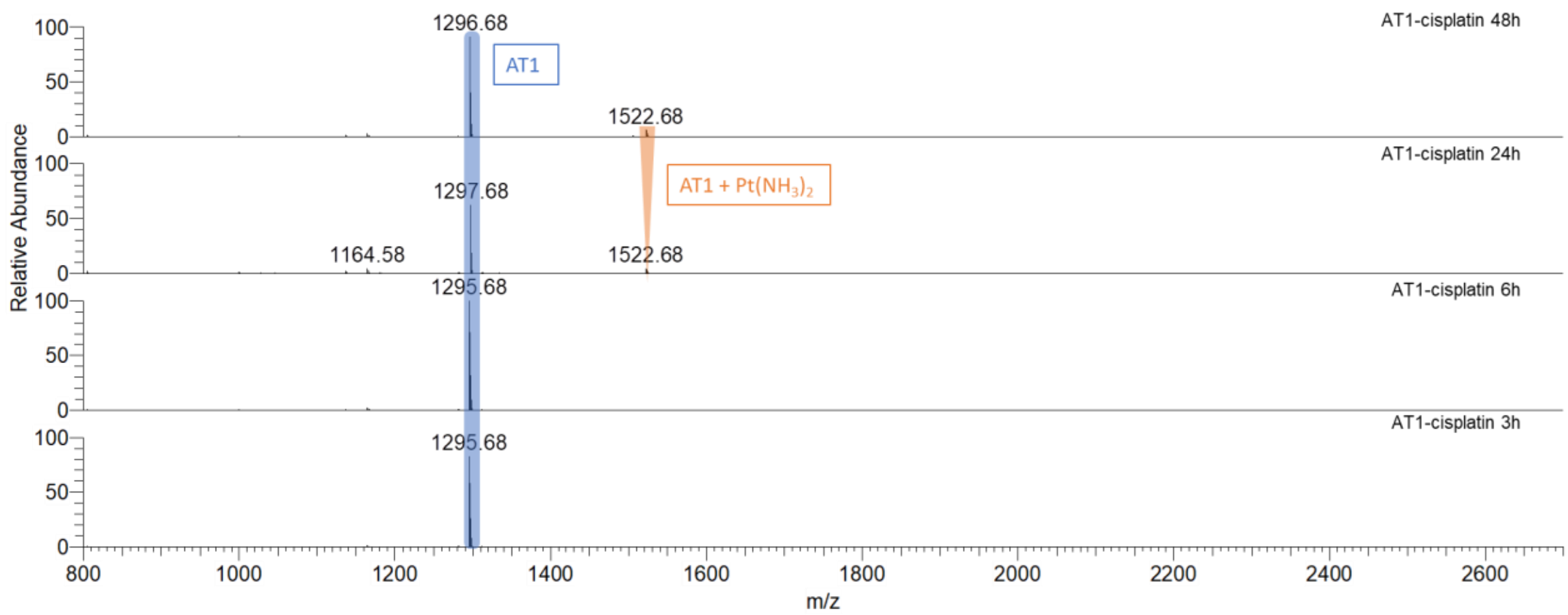


Figure S3 Time-resolved deconvoluted HR-ESI mass spectra for the incubation reaction of DDP with AT1 (3:1)

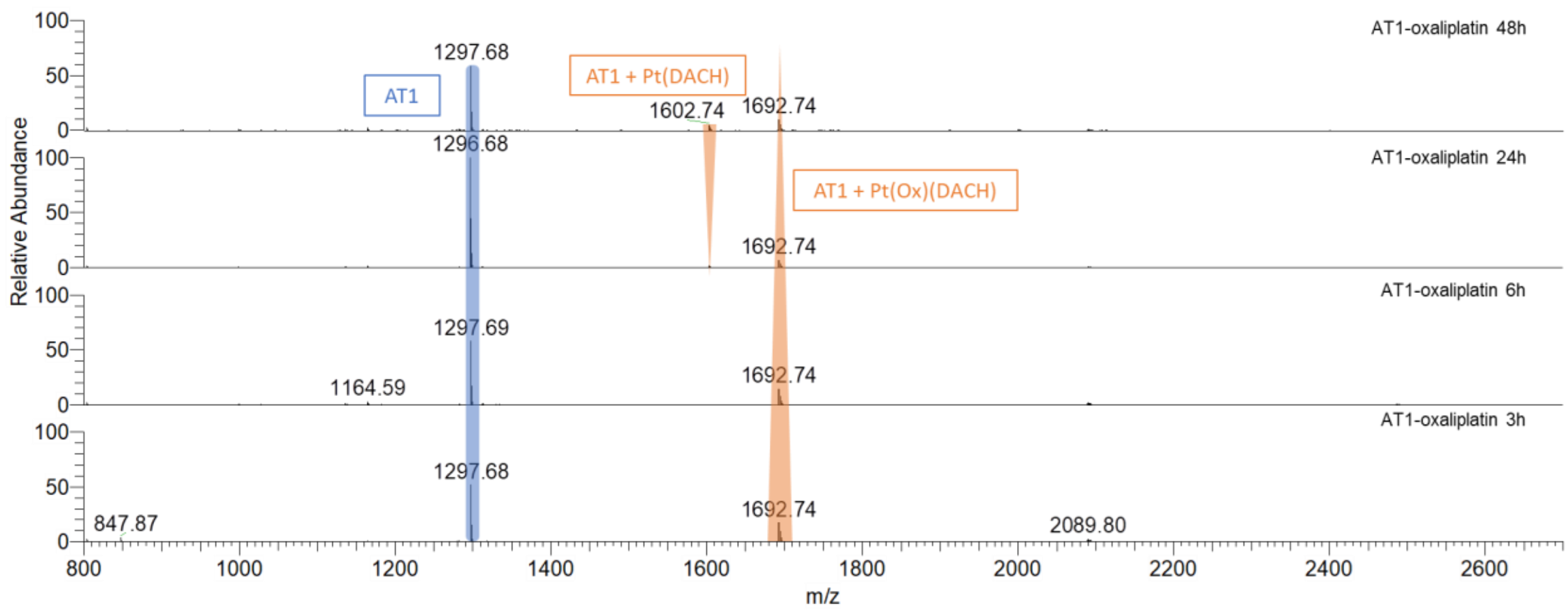


Figure S4 Time-resolved deconvoluted HR-ESI mass spectra for the incubation reaction of OxPt with AT1 (3:1)

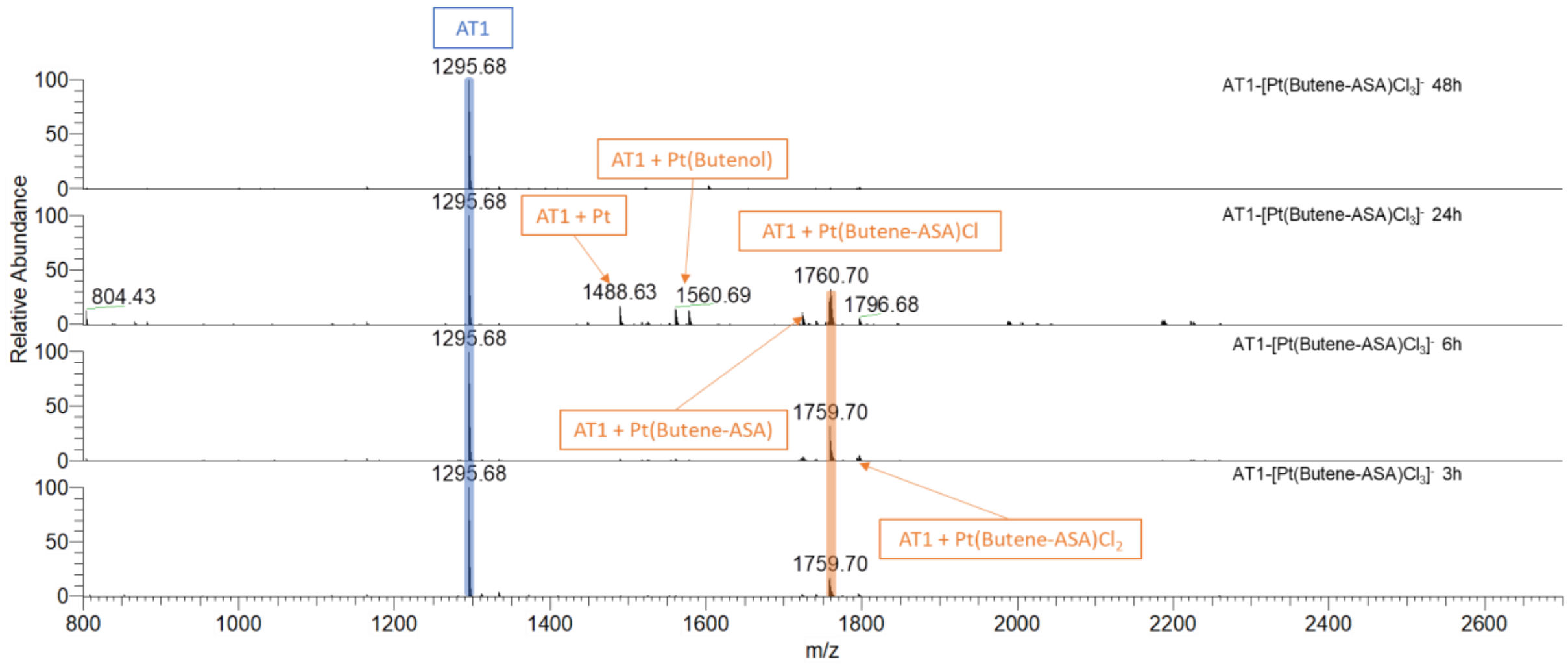


Figure S5 Time-resolved deconvoluted HR-ESI mass spectra for the incubation reaction of **1** with AT1 (3:1). The disappearance of adduct signals after 48 h may be due to aggregate formation and subsequent removal during sample filtration prior to MS analysis.

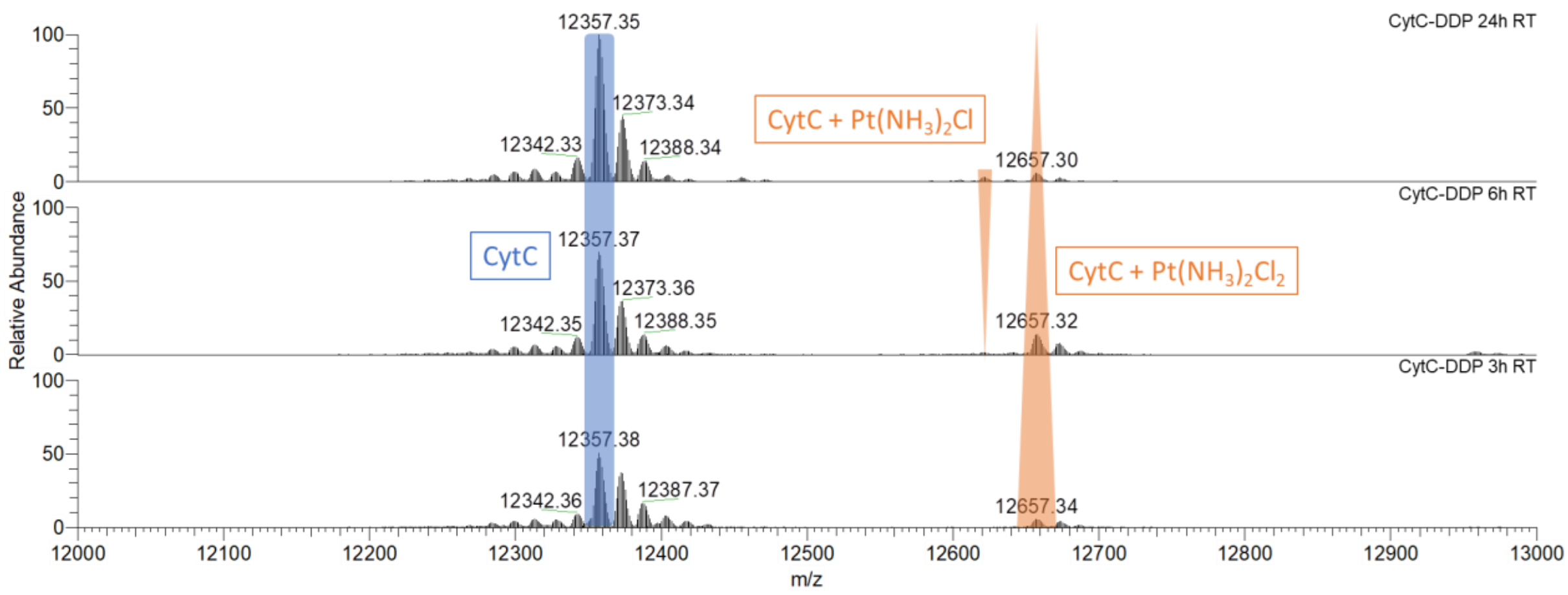


Figure S6 Time-resolved deconvoluted HR-ESI mass spectra for the incubation reaction of DDP with CytC at room temperature.

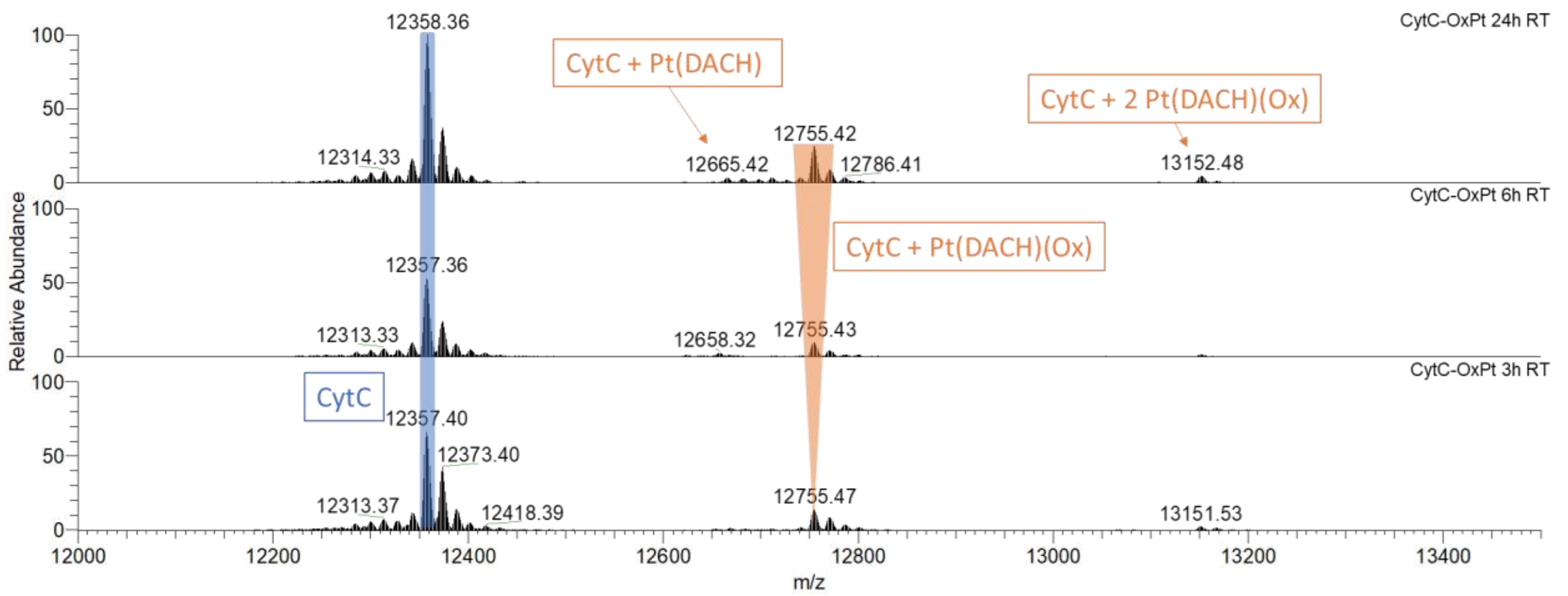


Figure S7 Time-resolved deconvoluted HR-ESI mass spectra for the incubation reaction of OxPt with CytC at room temperature.

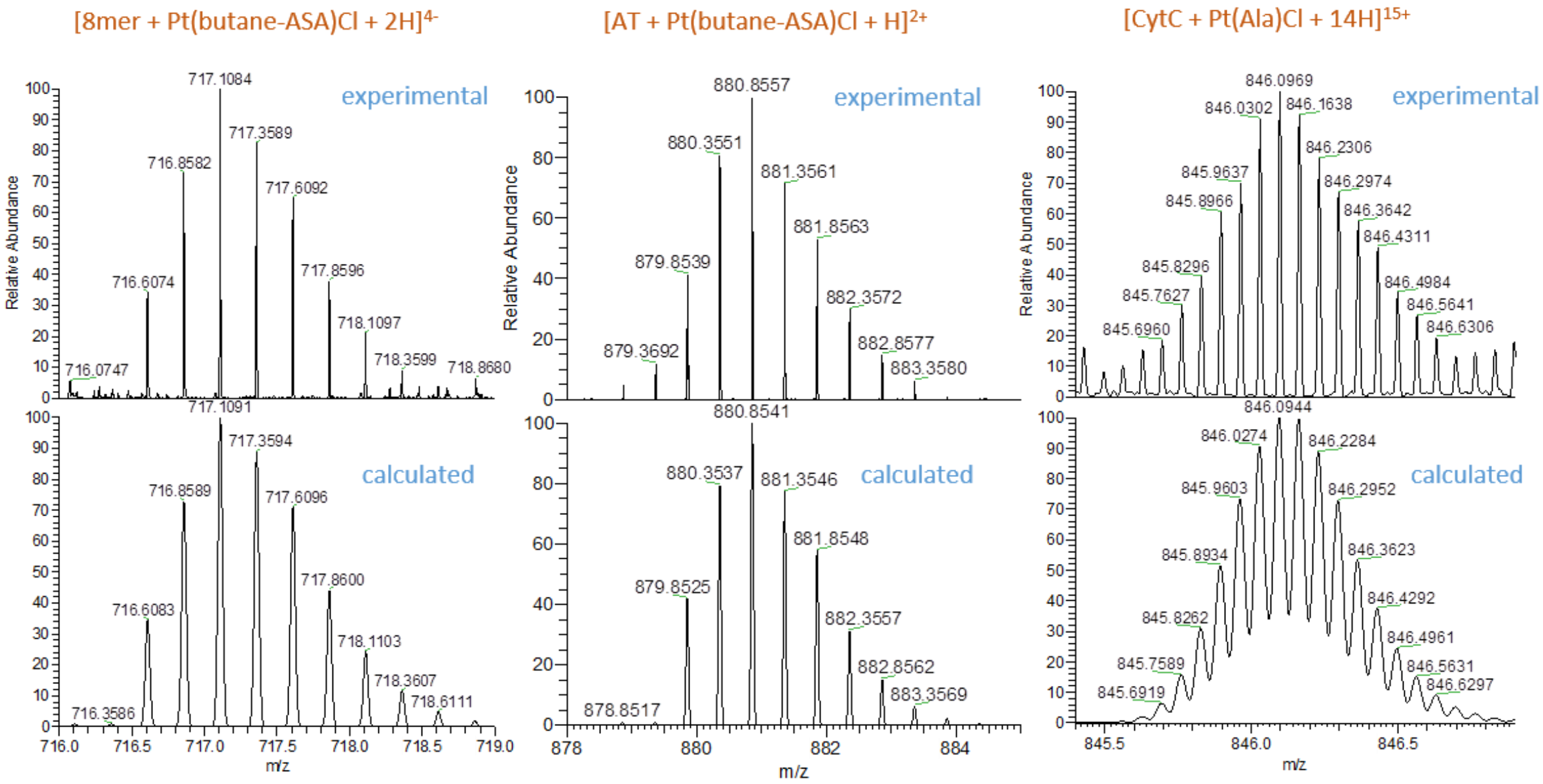


Figure S8 Examples of isotopic distributions for adducts of compound **2** on all three biomolecules.

## MS/MS

The extracted MS² spectra were uploaded to the Aom²S (in case of 8mer) or Apm²S (in case of AT1).[1] As possible ionizations, the loss/addition of up to 15 protons was imposed ((H+)-1--15,(H+)1-15,). As "first list of groups", "(Pt++)1-n" was entered for obtaining only the platinated fragments (with n being the maximum number of platinum ions found on the precursor ion). To obtain also non-platinated fragments, a comma has been added at the end. In the case of the Butene-ASA ligand, the degradation product Butene-SA was also considered giving the following enter: "(C13H14O4)1-n,(C11H12O3)1-n,", where n is the largest number of olefinic ligand found on the precursor ion.

Table S16 Apm²S and Aom²S search parameters

| Section | Parameter | Value |
|---|---|---|
| Spectrum Filters | Threshold | 1 % |
| | Mass accuracy | 5 ppm |
| | "Monoisotopic peak has to be present" | √ |
| | Minimum similarity | 80 % |
| | Zone | -0,5 – 3,5 |
| | Theoretical threshold | 1% |
| | Nb peaks limit | 4 |
| MS/MS fragments types (Aom²S) | Fragments | All |
| | Fragment losses | All |
| | Internal fragments | All |
| Peptidic sequence (Apm²S) | Fragmentation | a, b, y |
| | | ya, yb, immonium ions |

## 8mer

Table S17 List of platinated fragments identified when fragmenting the adduct $[8mer + Pt(NH_3)_2]^{5-}$ at m/z 526. N = nucleotide, n = nucleobase, f = methylene furan.

| Sequence | Type | $m/z_{calc}$ | $m/z_{exp}$ | Error [ppm] | Rel. Ab. [%] |
|---|---|---|---|---|---|
| g | $[Pt(NH_3) + g - 2 H]^{-}$ | 360.0178 | 360.0171 | -1.9 | 3.5 |
| ATTGGCAC | $[8mer + Pt(NH_3)]^{5-}$ | 522.8781 | 522.8774 | -1.3 | 10.1 |
| ATTGGCAC | $[8mer + Pt(NH_3)_2]^{5-}$ | 526.2834 | 526.2826 | -1.5 | 20.5 |
| TTGGCAC | $[w7\ A^* + Pt(NH_3)_2 + H]^{4-}$ | 566.0696 | 566.0689 | -1.2 | 1.2 |
| TTGGCAC | $[w7 + Pt(NH_3) + 2 H]^{4-}$ | 595.5766 | 595.5759 | -1.2 | 3.8 |
| TTGGCAC | $[w7 + Pt(NH_3)_2 + 2 H]^{4-}$ | 599.8332 | 599.8325 | -1.2 | 6.6 |
| ATTGGCAC | $[8mer\ A^* + Pt(NH_3)]^{4-}$ | 620.0858 | 620.0850 | -1.3 | 5.6 |
| ATTG | $[b4\ G^* + Pt(NH_3) - H]^{2-}$ | 621.5714 | 621.5705 | -1.4 | 1.4 |
| TTGf | $[y7{:}a5\text{-}G + Pt(NH_3) - H]^{2-}$ | 621.5714 | 621.5705 | -1.4 | 1.4 |
| ATTGGCAC | $[8mer\ A^* + Pt(NH_3)_2]^{4-}$ | 624.3425 | 624.3415 | -1.6 | 9.9 |
| ATTGGCAC | $[8mer\ T^* + Pt(NH_3)_2]^{4-}$ | 626.5953 | 626.5944 | -1.4 | 2.3 |
| ATTG | $[a4 + Pt(NH_3) - 2 H]^{2-}$ | 689.0986 | 689.0976 | -1.5 | 1.8 |
| TGGCAC | $[w6 + Pt(NH_3) + 2 H]^{3-}$ | 692.7554 | 692.7542 | -1.7 | 2.1 |
| TGGCAC | $[w6 + Pt(NH_3)_2 + 2 H]^{3-}$ | 698.7647 | 698.7636 | -1.6 | 1.5 |
| ATTGGCf | $[a7\ A^* + Pt]^{3-}$ | 718.7552 | 718.7540 | -1.7 | 1.2 |
| ATTGGCf | $[a7\ A^* + Pt(NH_3)]^{3-}$ | 724.4307 | 724.4295 | -1.7 | 6.1 |
| ATTGGCf | $[a7\ A^* + Pt(NH_3)_2 + H]^{3-}$ | 730.1062 | 730.1051 | -1.5 | 5.0 |
| TTGG | $[w7{:}a5 + Pt(NH_3)]^{2-}$ | 737.0792 | 737.0782 | -1.4 | 1.9 |
| TTGGCAC | $[y7 + Pt(NH_3) + 2 H]^{3-}$ | 767.7825 | 767.7810 | -2.0 | 1.4 |
| TGGCf | $[w6{:}a7\text{-}A + Pt(NH_3) + H]^{2-}$ | 818.5809 | 818.5796 | -1.6 | 2.0 |
| ATTGG | $[a5 + Pt(NH_3) - H]^{2-}$ | 854.1255 | 854.1239 | -1.9 | 1.7 |
| TTGGCf | $[w7{:}a7\text{-}A + Pt + 2 H]^{2-}$ | 962.5914 | 962.5892 | -2.3 | 1.5 |
| TTGGCf | $[w7{:}a7\text{-}A + Pt(NH_3) + 2 H]^{2-}$ | 971.1047 | 971.1025 | -2.3 | 4.3 |
| TTGGCf | $[w7{:}a7\text{-}A + Pt(NH_3)_2 + 2 H]^{2-}$ | 979.1173 | 979.1150 | -2.3 | 2.6 |
| TTG | $[w7{:}a4 + Pt(NH_3)]^{-}$ | 1146.1132 | 1146.1106 | -2.3 | 1.2 |
| TGG | $[w6{:}a5 + Pt(NH_3)]^{-}$ | 1171.1196 | 1171.1170 | -2.2 | 1.1 |

Table S18 List of platinated fragments identified when fragmenting the adduct $[8mer + 2\ Pt(NH_3)_2]^{3-}$ at m/z 953

| Sequence | Type | $m/z_{calc}$ | $m/z_{exp}$ | Error [ppm] | Rel. Ab. [%] |
|---|---|---|---|---|---|
| ga | $[Pt + g + a - H]^{-}$ | 478.0457 | 478.0449 | -1.7 | 1.1 |
| ga | $[Pt(NH_3) + g + a - H]^{-}$ | 495.0722 | 495.0715 | -1.4 | 1.3 |
| GCAC | $[w4 + Pt(NH_3)_2 + H]^{2-}$ | 731.6006 | 731.5995 | -1.5 | 1.1 |
| ATTGf | $[a5\ G^* + Pt(NH_3) - H]^{2-}$ | 778.1002 | 778.0987 | -1.9 | 1.6 |
| ATTGf | $[a5\ G^* + Pt(NH_3)_2 - H]^{2-}$ | 786.6134 | 786.6119 | -1.9 | 1.6 |
| ATTGGCAC | $[8mer + Pt + Pt(NH_3)]^{3-}$ | 936.4513 | 936.4495 | -1.9 | 4.9 |
| ATTGGCAC | $[8mer + 2\ Pt(NH_3)]^{3-}$ | 942.1268 | 942.1246 | -2.3 | 15.6 |
| ATTGGCAC | $[8mer + Pt(NH_3) + Pt(NH_3)_2]^{3-}$ | 947.8023 | 947.8003 | -2.1 | 35.9 |
| ATTGGCAC | $[8mer + 2\ Pt(NH_3)_2]^{3-}$ | 953.4778 | 953.4753 | -2.6 | 54.5 |

| | | | | | |
|---|---|---|---|---|---|
| ATTGG | [a5 + 2 $Pt(NH_3)$ - 3 H]$^{2-}$ | 958.6123 | 958.6100 | -2.4 | 1.4 |
| ATTGGCf | [a7 A* + Pt + $Pt(NH_3)$ - H]$^{2-}$ | 1183.6239 | 1183.6205 | -2.9 | 1.1 |
| ATTGGCf | [a7 A* + 2 $Pt(NH_3)$ - H]$^{2-}$ | 1192.6380 | 1192.6349 | -2.6 | 1.6 |
| ATTGGCA | [a7 + Pt + $Pt(NH_3)$ - H]$^{2-}$ | 1251.1511 | 1251.1480 | -2.5 | 3.1 |
| ATTGGCA | [a7 + 2 $Pt(NH_3)$ - H]$^{2-}$ | 1260.1653 | 1260.1622 | -2.5 | 5.4 |
| ATTG | [a4 + $Pt(NH_3)$ - H]$^{-}$ | 1379.2045 | 1379.2014 | -2.2 | 1.4 |

Table S19 List of platinated fragments identified when fragmenting the adduct [8mer + Pt(DACH) + H]$^{4-}$ at m/z 678.

| Sequence | Type | $m/z_{calc}$ | $m/z_{exp}$ | Error [ppm] | Rel. Ab. [%] |
|---|---|---|---|---|---|
| g | [Pt(DACH) + g - 2 H]$^{-}$ | 457.1070 | 457.1064 | -1.3 | 2.9 |
| ag | [Pt(DACH) + g + a - H]$^{-}$ | 592.1615 | 592.1611 | -0.7 | 7.4 |
| gg | [Pt(DACH) + 2 g - H]$^{-}$ | 608.1564 | 608.1560 | -0.7 | 1.2 |
| ATTGGCAC | [8mer A* + Pt(DACH)]$^{4-}$ | 644.3581 | 644.3573 | -1.2 | 3.5 |
| ATTGGCAC | [8mer + Pt(DACH) + H]$^{4-}$ | 678.1218 | 678.1207 | -1.6 | 18.2 |
| TTGf | [w7:a5-G + Pt(DACH)]$^{2-}$ | 710.0991 | 710.0981 | -1.4 | 1.1 |
| ATTGGC | [c6 + Pt(DACH)]$^{3-}$ | 724.4486 | 724.4464 | -3.0 | 1.5 |
| TTGGCA | [w7:a7 + Pt(DACH) + H]$^{3-}$ | 724.4486 | 724.4464 | -3.0 | 1.5 |
| ATTG | [a4 + Pt(DACH) - 2 H]$^{2-}$ | 737.6432 | 737.6423 | -1.2 | 3.2 |
| ATTGGCf | [a7 A* + Pt(DACH)]$^{3-}$ | 757.1276 | 757.1262 | -1.8 | 5.4 |
| ATTGGCA | [a7 + Pt(DACH)]$^{3-}$ | 802.1458 | 802.1441 | -2.1 | 1.8 |
| TTGGCAC | [w7 + Pt(DACH) + 3 H]$^{3-}$ | 826.8010 | 826.7997 | -1.6 | 4.4 |
| ATTGG | [a5 + Pt(DACH) - H]$^{2-}$ | 902.1695 | 902.1681 | -1.6 | 4.0 |
| ATTGGf | [a6 C* + Pt(DACH)]$^{2-}$ | 991.1710 | 991.1691 | -1.9 | 1.2 |
| TGGCAC | [w6 + Pt(DACH) + 3 H]$^{2-}$ | 1088.6820 | 1088.6795 | -2.3 | 1.3 |
| TTf | [w7:a4-G + Pt(DACH)]$^{-}$ | 1092.1530 | 1092.1510 | -1.8 | 1.8 |
| TTGGCAC | [y7 + Pt(DACH) + 3 H]$^{2-}$ | 1200.7220 | 1200.7193 | -2.2 | 1.4 |
| ATTf | [a4 G* + Pt(DACH) - H]$^{-}$ | 1325.2443 | 1325.2419 | -1.8 | 2.2 |
| TTGf | [y7:a5-G + Pt(DACH)]$^{-}$ | 1341.2392 | 1341.2367 | -1.9 | 1.1 |

Table S20 List of platinated fragments identified when fragmenting the adduct [8mer + 2 Pt(DACH) - H]$^{4-}$ at m/z 754

| Sequence | Type | $m/z_{calc}$ | $m/z_{exp}$ | Error [ppm] | Rel. Ab. [%] |
|---|---|---|---|---|---|
| ag | [Pt(DACH) + g + a - H]$^{-}$ | 592.1615 | 592.1609 | -1.0 | 4.9 |
| ATTGGCAC | [8mer + Pt(DACH) + H]$^{4-}$ | 678.1218 | 678.1205 | -1.9 | 3.8 |
| ATTG | [a4 + Pt(DACH) - 2 H]$^{2-}$ | 737.6432 | 737.6421 | -1.5 | 1.2 |
| ATTGGCAC | [8mer + 2 Pt(DACH) - H]$^{4-}$ | 754.8879 | 754.8865 | -1.9 | 4.0 |
| ATTGGCf | [a7 A* + Pt(DACH)]$^{3-}$ | 757.1276 | 757.1256 | -2.6 | 2.0 |
| TTGGCAC | [w7 + Pt(DACH) + 3 H]$^{3-}$ | 826.8010 | 826.7999 | -1.3 | 1.6 |
| ATTGGCf | [a7 A* + 2 Pt(DACH) - 2 H]$^{3-}$ | 859.4824 | 859.4801 | -2.7 | 1.8 |
| ATTGG | [a5 + Pt(DACH) - H]$^{2-}$ | 902.6702 | 902.6683 | -2.1 | 1.1 |
| ATTGGCA | [a7 + 2 Pt(DACH) - 2 H]$^{3-}$ | 904.5006 | 904.4978 | -3.1 | 5.2 |

| | | | | | |
|---|---|---|---|---|---|
| TTGGCAC | [w7 + 2 Pt(DACH) + H]$^{3-}$ | 929.1558 | 929.1538 | -2.2 | 1.1 |
| ATTGG | [a5 + 2 Pt(DACH) - 3 H]$^{2-}$ | 1056.2024 | 1056.1997 | -2.6 | 1.5 |
| TTf | [w7:a4-G + Pt(DACH)]$^{-}$ | 1092.1530 | 1092.1504 | -2.4 | 1.6 |
| GCf | [w4:a7-A + Pt(DACH)]$^{-}$ | 1102.1597 | 1102.1572 | -2.3 | 1.4 |
| ATTf | [a4 G* + Pt(DACH) - H]$^{-}$ | 1325.2441 | 1325.2414 | -2.0 | 2.4 |
| TTGf | [w7:a5-G + Pt(DACH) + H]$^{-}$ | 1421.2055 | 1421.2033 | -1.5 | 1.3 |
| ATTGf | [a5 G* + Pt(DACH)]$^{-}$ | 1654.2968 | 1654.2940 | -1.7 | 1.0 |

Table S21 List of platinated fragments identified when fragmenting the adduct [8mer + Pt(Butene-ASA)]$^{5-}$ at m/z 566 (from compound **1**). Fragments highlighted in yellow are identified after imposing a threshold of 0.5%.

| Sequence | Type | $m/z_{calc}$ | $m/z_{exp}$ | Error [ppm] | Rel. Ab. [%] |
|---|---|---|---|---|---|
| ATTGGCAC | [8mer + Pt]$^{5-}$ | 519.4728 | 519.4719 | -1.7 | 5.6 |
| ATTGGCAC | [8mer + Pt(Butene-ASA)]$^{5-}$ | 566.2907 | 566.2899 | -1.4 | 14.0 |
| ATTGGCAC | [8mer A* + Pt + H]$^{4-}$ | 615.5788 | 615.5778 | -1.6 | 1.5 |
| ATTGGCA | [w7 + Pt + 2 H]$^{4-}$ | 591.3200 | 591.3192 | -1.4 | 0.8 |
| ATTGGCAC | [8mer A* + Pt(Butene-ASA)]$^{4-}$ | 674.3515 | 674.3503 | -1.8 | 1.8 |
| ATTGGCAC | [8mer T* + Pt(Butene-ASA)]$^{4-}$ | 676.6044 | 676.6033 | -1.6 | 3.3 |
| ATTG | [a4 + Pt - 2 H]$^{2-}$ | 680.5853 | 680.5843 | -1.5 | 0.6 |
| ATTGGCA | [a7 A* + Pt]$^{3-}$ | 719.0890 | 719.0877 | -1.8 | 1.2 |
| ATTGGCA | [a7 + Pt + H]$^{3-}$ | 763.7732 | 763.7712 | -2.7 | 2.1 |
| ATTGGC | [c6 + Pt(Butene-ASA)]$^{3-}$ | 764.4399 | 764.4389 | -1.3 | 1.4 |
| TTGGCA | [w7:a7 + Pt(Butene-ASA) + H]$^{3-}$ | 764.4399 | 764.4389 | -1.3 | 1.4 |
| ATTGGCA | [a7 A* + Pt(Butene-ASA)]$^{3-}$ | 796.7849 | 796.7835 | -1.8 | 1.4 |
| ATTGG | [a5 + Pt - H]$^{2-}$ | 845.6123 | 845.6107 | -1.9 | 0.7 |

Table S22 List of platinated fragments identified when fragmenting the adduct [8mer + Pt(Butene-ASA)]$^{5-}$ at m/z 566 (from compound **2**). Fragments highlighted in yellow are identified after imposing a threshold of 0.5%.

| Sequence | Type | $m/z_{calc}$ | $m/z_{exp}$ | Error [ppm] | Rel. Ab. [%] |
|---|---|---|---|---|---|
| ATTGGCAC | [8mer + Pt]$^{5-}$ | 519.4728 | 519.4719 | -1.7 | 3.8 |
| ATTGGCAC | [8mer + Pt(Butene-ASA)]$^{5-}$ | 566.2907 | 566.2898 | -1.6 | 25.2 |
| ATTGGCA | [a7 + Pt - H]$^{4-}$ | 572.5782 | 572.5773 | -1.6 | 0.5 |
| TTGGCAC | [w7 + Pt + 2 H]$^{4-}$ | 591.0696 | 591.0688 | -1.4 | 1.0 |
| ATTGGCAC | [8mer A* + Pt]$^{4-}$ | 615.8292 | 615.8280 | -1.9 | 1.0 |
| ATTGGCA | [a7 + Pt(Butene-ASA) - H]$^{4-}$ | 631.3509 | 631.3499 | -1.6 | 1.5 |
| ATTGGCAC | [8mer G* + Pt(Butene-ASA)]$^{4-}$ | 670.3528 | 670.3519 | -1.3 | 1.7 |
| ATTGGCAC | [8mer A* + Pt[Butene-ASA)]$^{4-}$ | 674.3515 | 674.3503 | -1.8 | 3.9 |
| ATTGGCAC | [8mer T* + Pt(Butene-ASA)]$^{4-}$ | 676.6044 | 676.6032 | -1.8 | 5.1 |
| ATTGGCA | [a7 A* + Pt]$^{3-}$ | 719.0890 | 719.0877 | -1.8 | 0.9 |
| ATTGGC | [c6 + Pt(Butene-ASA)]$^{3-}$ | 764.4399 | 764.4384 | -2.0 | 1.9 |
| TTGGCA | [w7:a7 + Pt(Butene-ASA) + H]$^{3-}$ | 764.4399 | 764.4384 | -2.0 | 1.9 |
| ATTGGCA | [a7 A* + Pt(Butene-ASA)]$^{3-}$ | 796.7849 | 796.7835 | -1.8 | 3.5 |

| ATTGG | [a5 + Pt(Butene-ASA) - H]$^{2-}$ | 962.6570 | 962.6546 | -2.5 | 0.6 |
|---|---|---|---|---|---|

Table S23 List of platinated fragments identified when fragmenting the adduct [AT1 + Pt(DACH) + H]$^{3+}$ at m/z 535.

| Sequence | Type | m/z$_{calc}$ | m/z$_{exp}$ | Error [ppm] | Rel. Ab. [%] |
|---|---|---|---|---|---|
| PFH | [a9y4 + Pt(DACH) - H]$^{2+}$ | 331.1324 | 331.1327 | 0.9 | 4.5 |
| PFH | [b9y4 + Pt(DACH) - H]$^{2+}$ | 345.1298 | 345.1302 | 1.2 | 9.8 |
| PFHL | [y4 + Pt(DACH) - H]$^{2+}$ | 410.6772 | 410.6777 | 1.2 | 4.2 |
| H | [a9y2 + Pt(DACH) - 2 H]$^{+}$ | 417.1362 | 417.1366 | 1.0 | 4.1 |
| RVYIH | [a6y9 + Pt(DACH) - H]$^{2+}$ | 474.7302 | 474.7308 | 1.3 | 4.9 |
| DRVYIH | [a6 + Pt - H]$^{2+}$ | 475.1859 | 475.1865 | 1.3 | 7.5 |
| HPFHL | [y5 + Pt(DACH) - H]$^{2+}$ | 479.2066 | 479.2072 | 1.3 | 1.9 |
| DRVYIHPFH | [a9 + Pt(DACH)]$^{3+}$ | 482.2250 | 482.2256 | 1.2 | 14.1 |
| RVYIH | [b6y9 + Pt(DACH) - H]$^{2+}$ | 488.7277 | 488.7282 | 1.0 | 2.1 |
| DRVYIHPFH | [b9 + Pt(DACH)]$^{3+}$ | 491.5566 | 491.5575 | 1.8 | 6.8 |
| DRVYIH | [a6 + Pt(DACH) - H]$^{2+}$ | 532.2437 | 532.2445 | 1.5 | 34.8 |
| DRVYIHPFHL | [AT1 + Pt(DACH) + H]$^{3+}$ | 535.5887 | 535.5893 | 1.1 | 100.0 |
| DRVYIH | [b6 + Pt(DACH) - H]$^{2+}$ | 546.2412 | 546.2419 | 1.3 | 20.4 |
| PFH | [a9y4 + Pt - 2 H]$^{+}$ | 547.1417 | 547.1422 | 0.9 | 3.7 |
| YIHPFHL | [y7 + Pt(DACH) - H]$^{2+}$ | 617.7811 | 617.7817 | 1.0 | 1.1 |
| DRVYIHPF | [a8 + Pt(DACH) - H]$^{2+}$ | 654.3044 | 654.3051 | 1.1 | 4.9 |
| DRVYIHPF | [b8 + Pt(DACH) - H]$^{2+}$ | 668.3018 | 668.3025 | 1.0 | 5.2 |
| FHL | [y3 + Pt(DACH) - 2 H]$^{+}$ | 723.2942 | 723.2951 | 1.2 | 2.0 |

Table S24 List of platinated fragments identified when fragmenting the adduct [AT1 + Pt(Butene-ASA)$Cl_2$ + 2 H]$^{2+}$ at m/z 898 (from the incubation reaction of AT1 with compound **1**).

| Sequence | Type | m/z$_{calc}$ | m/z$_{exp}$ | Error [ppm] | Rel. Ab. [%] |
|---|---|---|---|---|---|
| DR | [a2 + Pt - 2 H]$^{+}$ | 437.0896 | 437.0905 | 2.1 | 1.8 |
| DR | [b2 + Pt - 2 H]$^{+}$ | 465.0845 | 465.0855 | 2.2 | 1.8 |
| DRV | [a3 + PtCl - H]$^{+}$ | 572.1346 | 572.1360 | 2.4 | 1.0 |
| DRV | [b3 + PtCl - H]$^{+}$ | 600.1296 | 600.1312 | 2.7 | 1.1 |
| DRVYIHPFH | [a9 + Pt - H]$^{2+}$ | 665.7760 | 665.7775 | 2.3 | 3.6 |
| DRVYIHPFH | [b9 + Pt - H]$^{2+}$ | 679.7734 | 679.7750 | 2.4 | 3.8 |
| DRVYIHPFH | [a9 + PtCl]$^{2+}$ | 683.7643 | 683.7659 | 2.3 | 4.6 |
| DRVYIHPFH | [b9 + PtCl]$^{2+}$ | 698.2619 | 698.2637 | 2.6 | 23.1 |
| DRVYIHPFHL | [AT1 + Pt]$^{2+}$ | 745.8215 | 745.8232 | 2.3 | 59.6 |
| DRVYIHPFHL | [AT1 + PtCl + H]$^{2+}$ | 763.8093 | 763.8110 | 2.2 | 69.8 |
| DRVYIHPFHL | [AT1 + $PtCl_2$ + 2 H]$^{2+}$ | 781.7973 | 781.7991 | 2.3 | 25.1 |
| DRVYIHPFHL | [AT1 + Pt(Butene-ASA)Cl + H]$^{2+}$ | 880.8541 | 880.8561 | 2.3 | 3.7 |
| DRVYIHPFHL | [AT1 + Pt(Butene-ASA)$Cl_2$ + 2 H]$^{2+}$ | 898.8421 | 898.8439 | 2.0 | 39.1 |
| DRVYIH | [a6 + Pt - 2 H]$^{+}$ | 949.3645 | 949.3665 | 2.1 | 1.4 |
| DRVYIH | [b6 + Pt - 2 H]$^{+}$ | 977.3594 | 977.3615 | 2.1 | 2.3 |
| DRVYIH | [b6 + PtCl - H]$^{+}$ | 1013.3361 | 1013.3381 | 2.0 | 4.7 |

Table S25 List of platinated fragments identified when fragmenting the adduct [AT1 + Pt(Butene-ASA)Cl + H]$^{2+}$ at m/z 880 (from the incubation reaction of AT1 with compound **1**).

| Sequence | Type | $m/z_{calc}$ | $m/z_{exp}$ | Error [ppm] | Rel. Ab. [%] |
|---|---|---|---|---|---|
| DR | $[a2 + Pt - 2 H]^{+}$ | 437.0896 | 437.0897 | 0.2 | 8.9 |
| DR | $[b2 + Pt - 2 H]^{+}$ | 465.0845 | 465.0847 | 0.4 | 3.7 |
| DRVYIH | $[a6 + PtCl]^{2+}$ | 493.1741 | 493.1743 | 0.4 | 1.8 |
| DRV | $[a3 + Pt - 2 H]^{+}$ | 536.1580 | 536.1584 | 0.7 | 3.4 |
| PFH | $[a9y4 + Pt - 2 H]^{+}$ | 547.1417 | 547.1419 | 0.4 | 1.1 |
| DRV | $[a3 + PtCl - H]^{+}$ | 572.1347 | 572.1352 | 0.9 | 2.8 |
| DRVYIHPF | $[a8 + Pt - H]^{2+}$ | 597.2465 | 597.2469 | 0.7 | 1.1 |
| RVYIHPFH | $[a9y9 + Pt - H]^{2+}$ | 608.2625 | 608.2629 | 0.7 | 1.1 |
| RVYIHPFH | $[b9y9 + PtCl]^{2+}$ | 640.7484 | 640.7489 | 0.8 | 1.6 |
| DRVYIHPFH | $[a9 + Pt - H]^{2+}$ | 665.7760 | 665.7764 | 0.6 | 11.7 |
| DRVYIHPFH | $[b9 + Pt - H]^{2+}$ | 679.7734 | 679.7739 | 0.7 | 9.0 |
| DRVYIHPFH | $[a9 + PtCl]^{2+}$ | 683.7643 | 683.7649 | 0.9 | 5.7 |
| RVYIHPFHL | $[y9 + Pt - H]^{2+}$ | 687.8071 | 687.8077 | 0.9 | 1.6 |
| DRVYIHPFH | $[b9 + PtCl]^{2+}$ | 698.2619 | 698.2626 | 1.0 | 18.7 |
| DRVY | $[b4 + Pt - 2 H]^{+}$ | 727.2164 | 727.2170 | 0.8 | 2.1 |
| DRVYIHPFHL | $[AT1 + Pt]^{2+}$ | 745.8215 | 745.8219 | 0.5 | 99.4 |
| DRVYIHPFHL | $[AT1 + PtCl + H]^{2+}$ | 763.8093 | 763.8097 | 0.5 | 69.4 |
| DRVYIHPFHL | $[AT1 + Pt(Butene\text{-}ASA)Cl + H]^{2+}$ | 880.8541 | 880.8546 | 0.6 | 4.3 |
| DRVYIH | $[a6 + Pt - 2 H]^{+}$ | 949.3645 | 949.3649 | 0.4 | 12.9 |
| DRVYIH | $[b6 + Pt - 2 H]^{+}$ | 977.3594 | 977.3599 | 0.5 | 5.7 |
| DRVYIH | $[b6 + PtCl - H]^{+}$ | 1013.3361 | 1013.3363 | 0.2 | 3.7 |

Table S26 List of platinated fragments identified when fragmenting the adduct [AT1 + Pt(Butene-ASA)Cl + H]$^{2+}$ at m/z 880 (from the incubation reaction of AT1 with compound **2**).

| Sequence | Type | $m/z_{calc}$ | $m/z_{exp}$ | Error [ppm] | Rel. Ab. [%] |
|---|---|---|---|---|---|
| DR | $[a2 + Pt - 2 H]^{+}$ | 437.0896 | 437.0902 | 1.4 | 26.8 |
| DR | $[b2 + Pt - 2 H]^{+}$ | 465.0845 | 465.0852 | 1.5 | 6.0 |
| DRVYIH | $[a6 + PtCl]^{2+}$ | 493.1742 | 493.1750 | 1.6 | 3.6 |
| DRV | $[a3 + Pt - 2 H]^{+}$ | 536.1580 | 536.1589 | 1.7 | 7.6 |
| PFH | $[a9y4 + Pt - 2 H]^{+}$ | 547.1417 | 547.1423 | 1.1 | 4.0 |
| YIH | $[a6y7 + Pt - 2 H]^{+}$ | 579.1679 | 579.1689 | 1.7 | 2.0 |
| DRVYIHPF | $[a8 + Pt - H]^{2+}$ | 597.2465 | 597.2475 | 1.7 | 3.2 |
| RVYIHPFH | $[a9y9 + Pt - H]^{2+}$ | 608.2625 | 608.2635 | 1.6 | 3.0 |
| RVYIHPFH | $[b9y9 + PtCl]^{2+}$ | 640.7484 | 640.7493 | 1.4 | 1.3 |
| DRVYIHPFH | $[a9 + Pt - H]^{2+}$ | 665.7760 | 665.7770 | 1.5 | 13.7 |
| DRVYIHPFH | $[b9 + Pt - H]^{2+}$ | 679.7734 | 679.7744 | 1.5 | 6.9 |
| DRVYIHPFH | $[a9 + PtCl]^{2+}$ | 683.7643 | 683.7653 | 1.5 | 3.6 |
| DRVYIHPFH | $[b9 + PtCl]^{2+}$ | 698.2619 | 698.2631 | 1.7 | 11.6 |
| DRVY | $[a4 + Pt - 2 H]^{+}$ | 699.2214 | 699.2225 | 1.6 | 5.5 |

| | | | | | |
|---|---|---|---|---|---|
| DRVY | [b4 + Pt - 2 H]$^{+}$ | 727.2164 | 727.2175 | 1.5 | 3.5 |
| DRVYIHPFHL | [AT1 + Pt]$^{2+}$ | 745.8215 | 745.8224 | 1.2 | 99.4 |
| DRVYIHPFHL | [AT1 + PtCl + H]$^{2+}$ | 763.8093 | 763.8101 | 1.0 | 85.3 |
| RVYIH | [a6y9 + Pt - 2 H]$^{+}$ | 834.3375 | 834.3386 | 1.3 | 1.4 |
| HPFHL | [y5 + Pt - 2 H]$^{+}$ | 843.2902 | 843.2912 | 1.2 | 1.6 |
| DRVYIHPFHL | [AT1 + Pt(Butene-ASA)Cl + H]$^{2+}$ | 880.8541 | 880.8555 | 1.6 | 7.2 |
| DRVYIH | [a6 + Pt - 2 H]$^{+}$ | 949.3645 | 949.3654 | 0.9 | 17.2 |
| YIHPFH | [a9y7 + Pt - 2 H]$^{+}$ | 960.3481 | 960.3503 | 2.3 | 1.1 |
| DRVYIH | [b6 + Pt - 2 H]$^{+}$ | 977.3594 | 977.3607 | 1.3 | 5.7 |
| DRVYIH | [b6 + PtCl - H]$^{+}$ | 1013.3361 | 1013.3372 | 1.1 | 3.7 |
| VYIHPFH | [a9y8 + Pt - 2 H]$^{+}$ | 1059.4166 | 1059.4178 | 1.1 | 1.1 |

## UV experiments

<table>
<tr><td>Blank</td><td>DDP</td><td>OxPt</td><td>2</td><td>1</td><td>Time (h)</td></tr>
<tr><td colspan="5">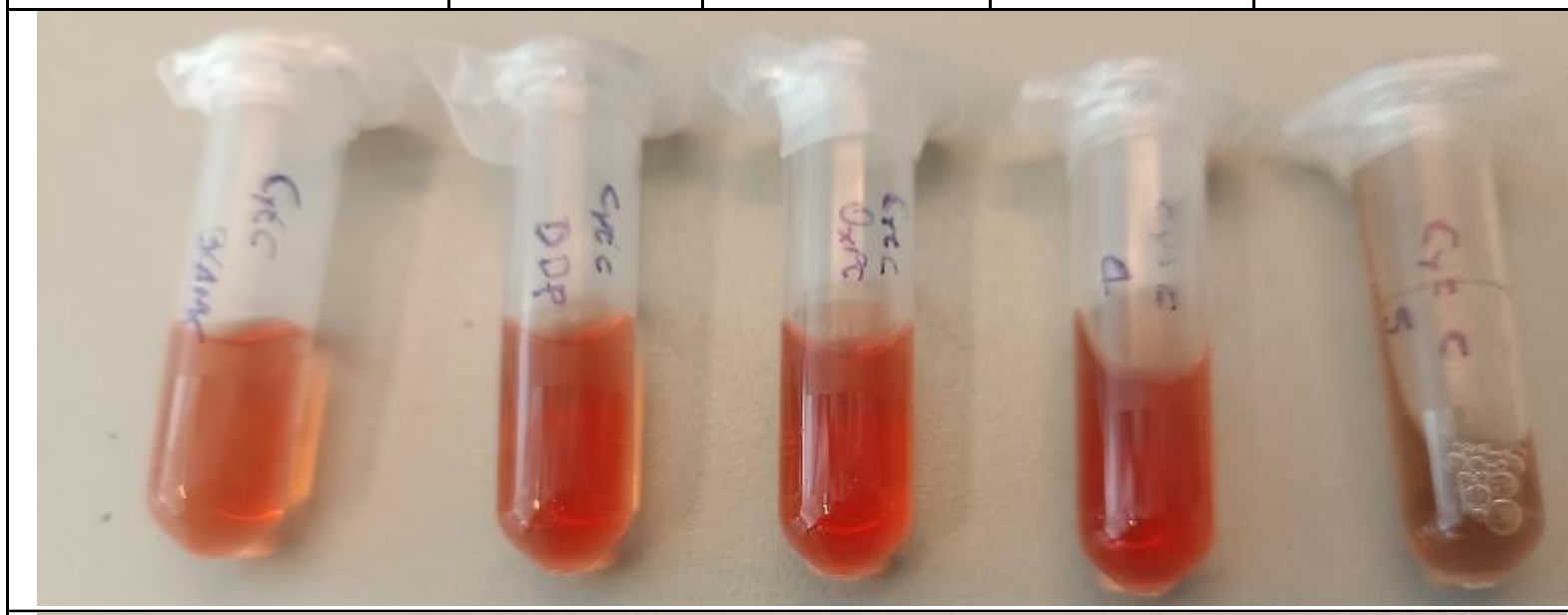</td><td>3</td></tr>
<tr><td colspan="5">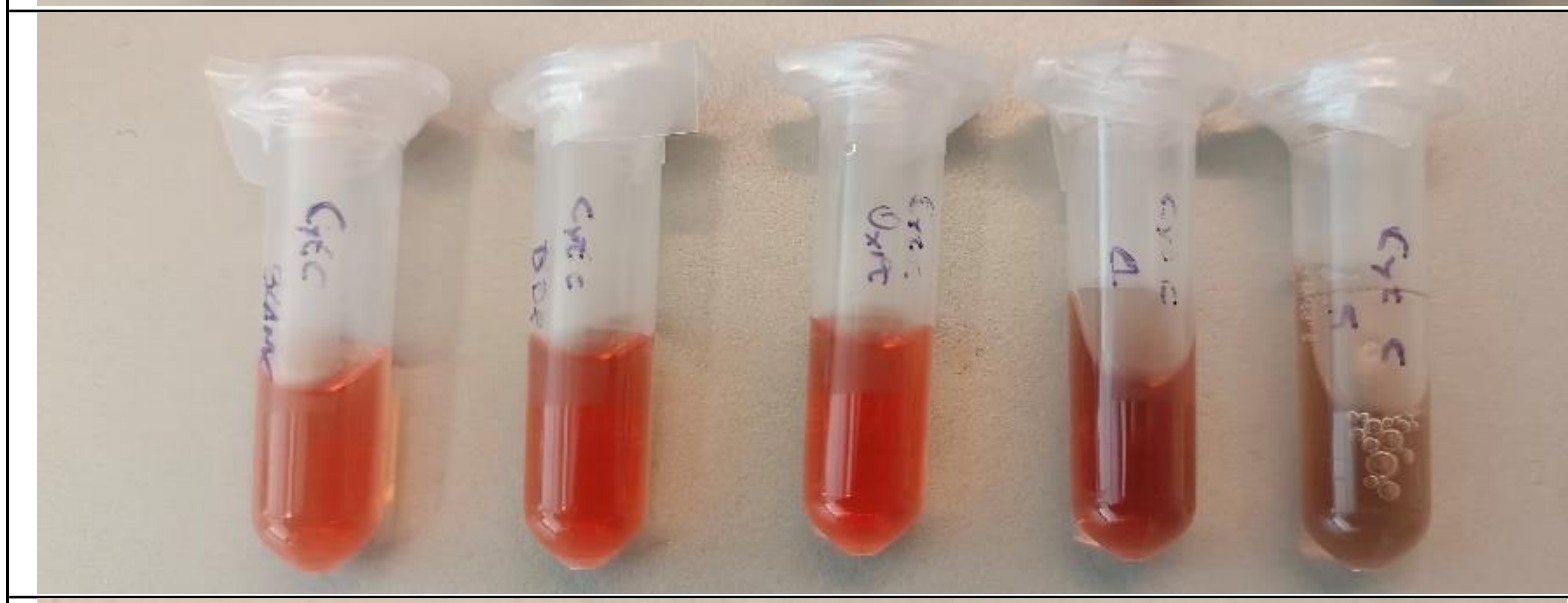</td><td>6</td></tr>
<tr><td colspan="5">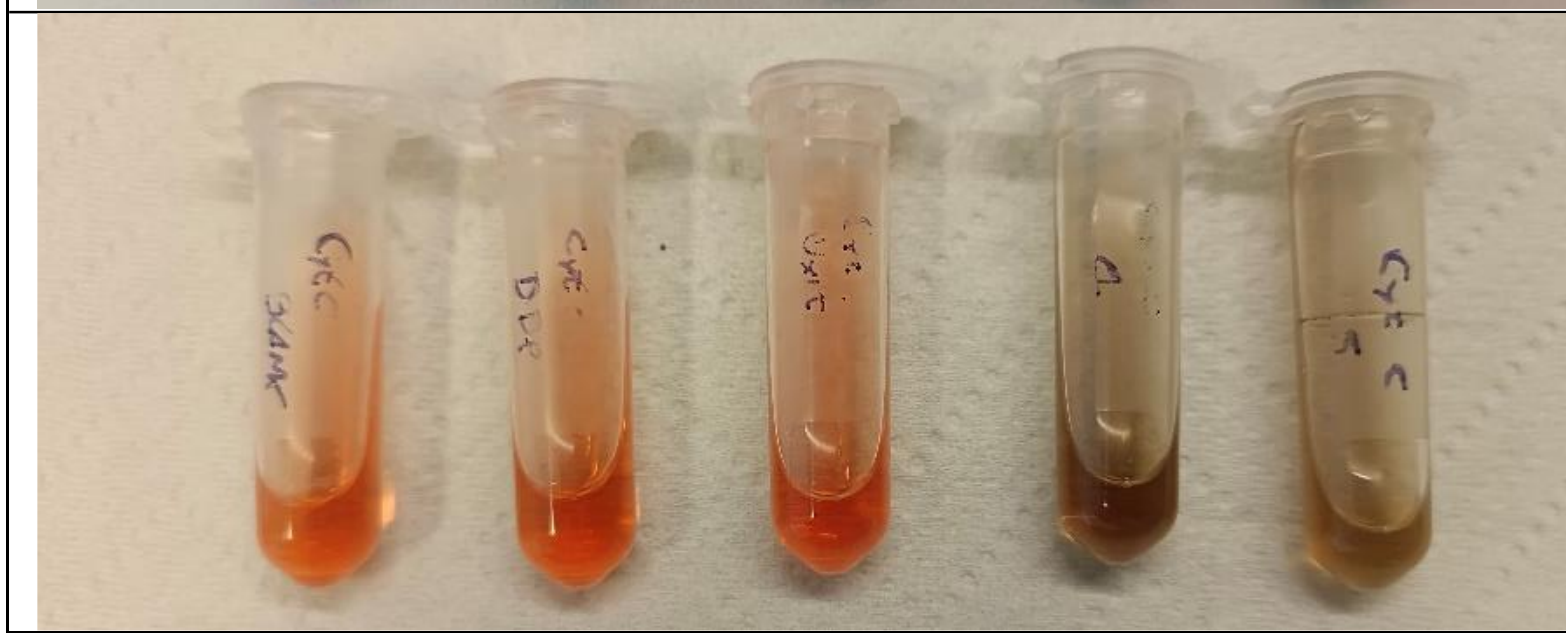</td><td>24</td></tr>
<tr><td colspan="5">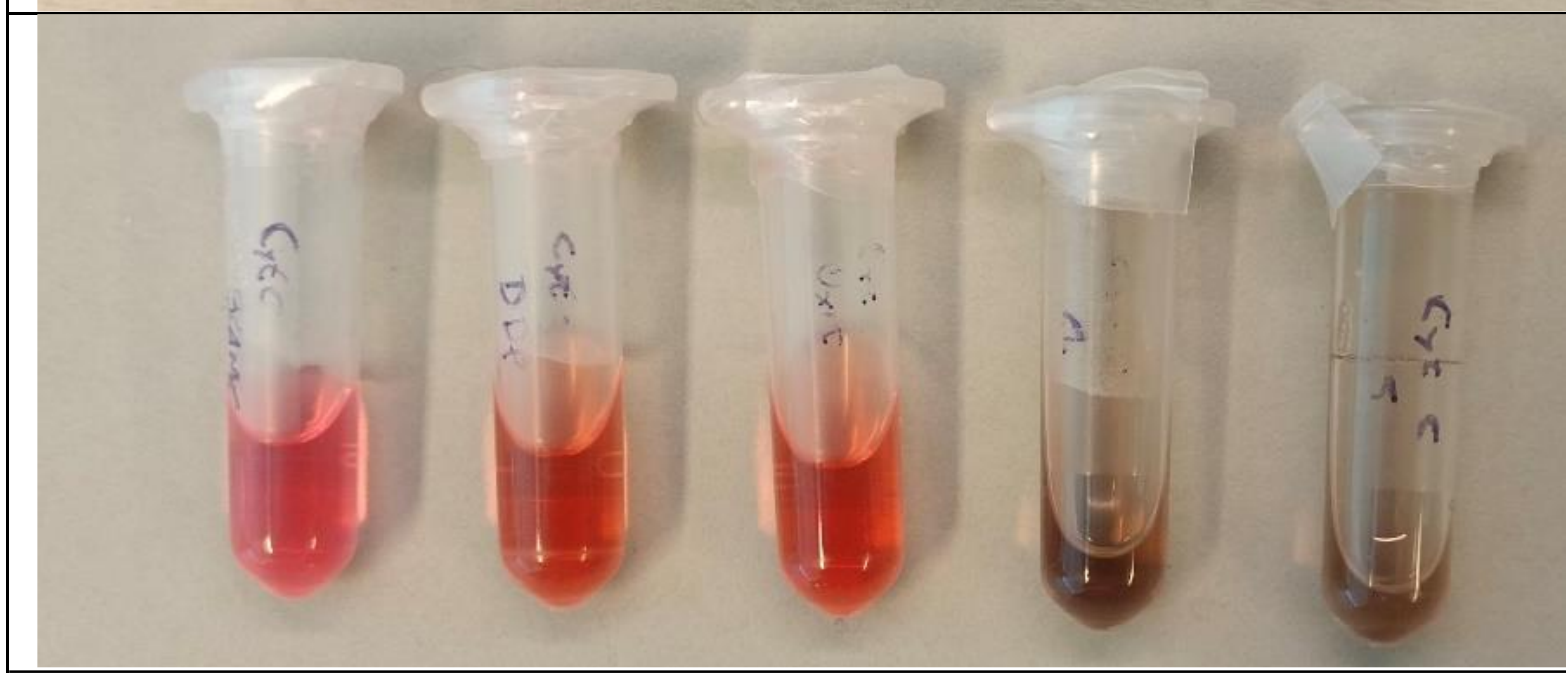</td><td>48</td></tr>
</table>

Figure S9 Time-resolved change in color of the incubation reaction's samples of CytC (blank) and CytC with DDP, OxPt, 2, and **1**

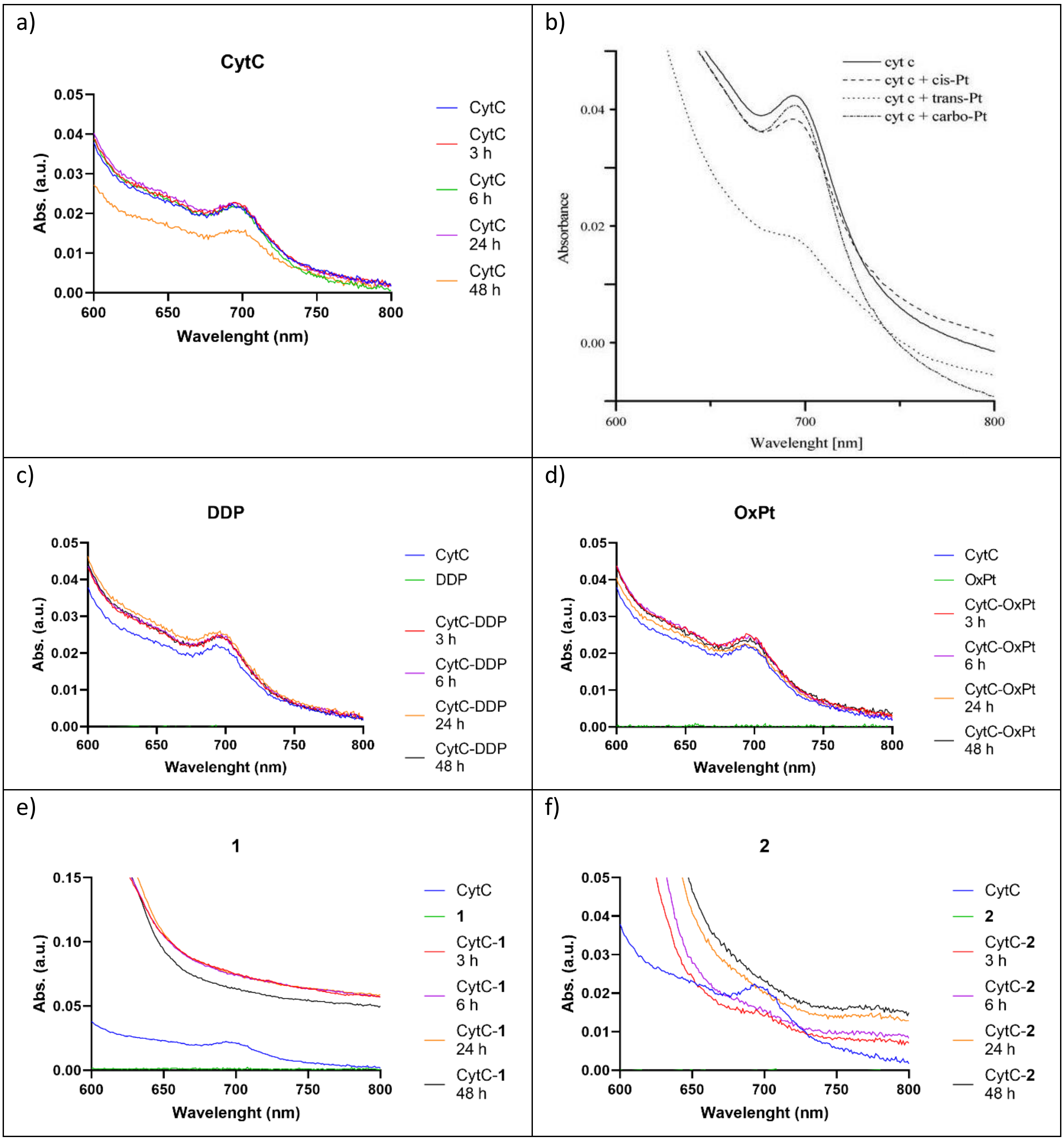


Figure S10 a) Time-resolved UV-Vis spectra of the ferricytochrome c; b) UV-Vis spectra of ferric CytC interacting with different Pt(II)-complexes as reported in the literature;[2] Time-resolved UV-Vis spectra of the incubation reaction of CytC with c) DDP; d) OxPt; e) **1**; f) **2**. All the spectra are zoomed in the wavelength range 600 – 800 nm to better appreciate the changes in the absorption band at 695 nm produced by the interaction Fe(III)-Met80.

## References:

[1] D. Ortiz, N. Gasilova, F. Sepulveda, L. Patiny, P. J. Dyson, and L. Menin, "Aom2S: A new web-based application for DNA/RNA tandem mass spectrometry data interpretation," *Rapid Communications in Mass Spectrometry*, vol. 34, no. 23, Dec. 2020, doi: 10.1002/rcm.8927.

[2] U. Śliwińska-Hill and L. Trynda-Lemiesz, "Effect of platinum anticancer drugs on the cytochrome c conformation," *Inorganica Chim. Acta*, vol. 457, pp. 130–135, Mar. 2017, doi: 10.1016/j.ica.2016.12.014.
